# Small Molecule‐Induced Differentiation As a Potential Therapy for Liver Cancer

**DOI:** 10.1002/advs.202103619

**Published:** 2022-03-27

**Authors:** Xu Zhang, Xiang‐Jie Zhu, Zhi Zhong, Jiang‐Chuan Du, Guo‐Xu Fang, Xiu‐liang Cui, Ling‐Ting Guan, Yan‐Yu Hu, Hong‐Yang Wang, Pei‐Lin Zhang

**Affiliations:** ^1^ National Center for Liver Cancer Shanghai 201805 China; ^2^ The International Cooperation Laboratory on Signal Transduction, Eastern Hepatobiliary Surgery Hospital Second Military Medical University Shanghai 200438 China; ^3^ Institute of Metabolism and Integrative Biology Fudan University Shanghai 200433 China; ^4^ Fudan University Shanghai Cancer Center, Department of Oncology, Shanghai Medical College Fudan University Shanghai 200032 China

**Keywords:** cancer heterogeneity, differentiation therapy, liver cancer, metabolic changes, small molecules

## Abstract

Despite the efficacy demonstrated by immunotherapy recently, liver cancer still remains one of the deadliest cancers, mainly due to heterogeneity of this disease. Continuous exploration of new therapeutics is therefore necessary. Chemical‐induced cell differentiation can serve as a promising approach, with its ability to consistently remodel gene expression profile and alter cell fate. Inspired by advances in stem cell and reprogramming field, here it is reported that a small molecule cocktail (SMC) consisted of: SB431542 (TGF*β* inhibitor), CHIR99021 (GSK3*β* inhibitor), BIX01294 (H3K9 methyltransferase/G9a inhibitor), and all‐trans retinoic acid (ATRA), can induce differentiation of liver cancer cells including cell lines, primary cancer cells, cancer stem cells, and drug resistant cells. Treated cells lose malignant characteristics and regain hepatocyte phenotype instead. When applied in vivo, SMC induces wide range of tissue necrosis or fibrosis within the tumors, while remaining tissues begin to express hepatic nuclear factor 4*α* (HNF4*α)*, the hepatic nuclear marker. SMC also leads to tumor abrogation in orthotopic xenograft models and life span extension of animals. The powerful differentiation induction of SMC is exerted through modulation of Akt/mTOR/HIF1*α* signaling and metabolic reprogramming, as well as suppressing Snail and enhancing HNF4*α* expression. Together, these results highlight that chemical‐induced differentiation has the potential to effectively treat liver cancer disregard of heterogeneity.

## Graphical abstract

A chemical cocktail can induce differentiation of liver cancer cells regardless of their heterogeneity through modulation of Akt/mTOR signaling, Snail and Warburg effect. Treated cells lose malignant characteristics and regain hepatocyte phenotype. When applied in vivo, this cocktail can lead to tumor abrogation and extension of life span of animals while not affecting normal liver cell behavior and regeneration.

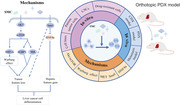

## 1 Introduction

Liver cancer is currently the third leading cause of cancer‐related deaths worldwide (second in male population). Till today, it still lacks effective treatment options.^[^ ^1^ ^]^ Given that causes and genetics of liver cancer vary widely among patients and largely undruggable, only a handful of targeted agents such as Sorafenib have been developed throughout the years, which brought modest clinical benefits accompanied with severe side effects.^[^ ^2^ ^]^ Recently, the systemic management of liver cancer has been revolutionized by the combination therapy of atezolizumab (programmed death‐ligand 1[PD‐L1] inhibitor) plus bevacizumab (vascular endothelial growth factor[VEGF] antibody), which significantly extended the overall survival for liver cancer patients, with median overall survival (mOS) reaching 19.2 months versus 13.6 months with Sorafenib.^[^ ^3^ ^]^ While this is an astounding achievement, compared to the ample treatment options for other tumors, novel therapies still need to be explored and developed.

“Differentiation therapy” was once a popular direction in anti‐cancer therapeutics research. It refers to using pharmacological intervention to reactivate the differentiation program of cancer cells, which can theoretically lead to their maturation along with loss of malignant phenotype.^[^ ^4^, ^5^, ^6^ ^]^ However, the only successful application of this theory so far was the use of all‐trans retinoic acid (ATRA) in the treatment of acute promyelocytic leukemia (APL).^[^ ^7^, ^8^ ^]^ This strategy has achieved little success on solid tumors, however, possibly due to higher inter‐ and intra‐tumor heterogeneity.^[^ ^4^, ^6^, ^9^, ^10^ ^]^ Nevertheless, over the years, differentiation strategies have been expanded to include cancer stem cell‐targeted, transcription factor‐based, and epigenetic‐associated strategies.^[^ ^10^ ^]^ For example, it was previously demonstrated that ectopic expression of the transcription factor hepatic nuclear factor 4*α* (HNF4*α*) could induce differentiation of liver cancer cells.^[^ ^11^ ^]^ Yet many differentiation strategies still faced various challenges, including limited efficacy, potential risks and operational complexity, thus, most studies in the field aimed to promote mechanistic understanding rather than clinical application.

In comparison to other induction agents such as transcription factor, small molecules offer more translational advantages. Inspired by advances in the field of cell differentiation and transdifferentiation (also known as lineage reprogramming) in recent years, we developed a small molecule cocktail (SMC) that could consistently induce differentiation of liver cancer cells regardless of their heterogeneous backgrounds and features. Under SMC treatment, part of liver cancer cells became apoptotic, while the rest of the cells lost their malignant features, and instead gained phenotype, functions and marker expression unique to mature hepatocyte. When applied in vivo, this cocktail also exhibited potent anti‐tumor capabilities on various xenograft animal models that mimic different types of liver cancer. It not only offers a new potential therapeutic strategy against this malignant tumor, but may also introduce a solution for cancer heterogeneity‐related issues, including drug resistance.

## 2 Results

### 2.1 Selection of Small Molecules to Induce Liver Cancer Cell Differentiation

Based on previous cell differentiation and transdifferentiation researches, we believe that to induce differentiation of liver cancer cells, a combination of molecules regulating signaling pathways, cell metabolism, cell epigenetics, and gene transcription is necessary. Previous studies have reported that transforming growth factor beta (TGF*β*) and glycogen synthase kinase 3 beta (GSK3*β*) /Wnt signaling pathways contribute to long term maintenance of hepatocyte's phenotype and functions,^[^ ^12^ ^]^ indicating that these signaling are involved in hepatocyte's master gene regulatory network (GRN). Since these two pathways are also involved in liver cancer initiation and progression, we screened through a cohort of candidates including signaling modulators SB431542, RepSox and LDN193189 (TGF*β* and bone morphogenetic protein[BMP] signaling regulators); signaling and metabolic modulators CHIR99021 and BIO (GSK3*β* signaling and cell metabolism); epigenetic modulators valproic acid (VPA) and azacitidine (5‐AZA); and transcription modulator ATRA, which was also the differentiation inducer of APL cells. Through screening, we initially found that SB431542 (S), CHIR99021(C), VPA (V), 5‐AZA (Z) and ATRA (A)—a 5‐factor cocktail (SCVZA) was able to suppress liver cancer cell HepG2's proliferation and change its tumor cell morphology. Other molecules targeting the same signal pathways, such as the TGF*β* modulator RepSox, while also exhibited efficacy, was not as efficient as SB431542, and therefore not chosen.

We used HNF4*α* as hepatic marker and clone formation ability as tumorigenicity marker to evaluate success of cancer cell differentiation. HNF4*α* not only is a master regulator of hepatic lineage differentiation and hepatic function maintenance, but a previous report also indicated that ectopic expression of HNF4*α* alone can induce differentiation of liver cancer cells.^[^ ^11^ ^]^ Under the treatment of SCVZA for 14 days, treated HepG2 cells exhibited profound morphological changes, increased HNF4*α* expressions and reduced colony formations (Figure S1A–C, Supporting Information). However, some colonies still emerged in treated samples (Figure S1C, Supporting Information) indicating incomplete differentiation. To further enhance differentiation efficiency, we added several other chemicals such as BIX01294, an epigenetic regulator with the ability to stabilize the hepatic phenotype, VitC, QNZ (NF‐kB inhibitor), and Brdu (epigenetic modulator and differentiation promoter) into the combination, and found that the addition of BIX01294 (B) and Brdu (Br) could significantly improve differentiation efficiency (Figure S1D–F, Supporting Information).

To determine the essential chemicals, we withdrew individual factor from the combination and found four chemicals: SB431542 (S), CHIR99021(C), BIX01294 (B), and ATRA (A) were essential for the induction of cancer cell differentiation (Figure S1G–I, Supporting Information). Under influence of these four factors, no colonies emerged in treated cell samples, implying complete loss of tumorigenicity potential (Figure S1I, Supporting Information). We then conducted systematic investigations using this 4‐factor small molecule cocktail (SMC).

### 2.2 Chemical Cocktail Induces Liver Cancer Cell Differentiation with the Loss of Malignant Characteristics

**Figure 1:**
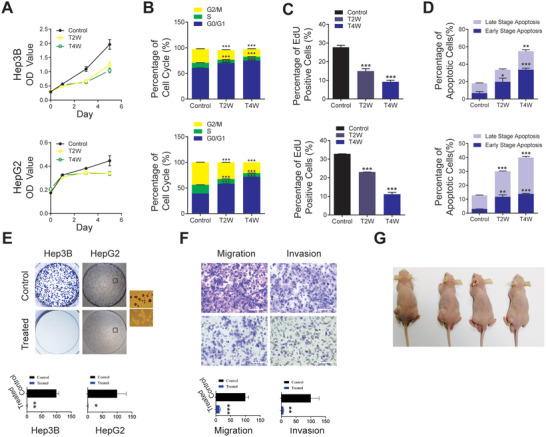
SMC treatment abrogates malignant features and tumorigenicity potential of liver cancer cells. A) Relative cell viability of control and SMC‐treated cells (*n* = 3). B) Cell cycle analysis using PI staining and flow cytometry (*n* = 3). C) FACS analysis of EdU incorporation rate of control and SMC‐treated cells (*n* = 3). D) FACS analysis of apoptotic population after SMC treatment measured with annexin V staining (*n* = 3). E) Plate colony formation and quantification (lower panel) (*n* = 3). Representative images (boxed areas) of HepG2 cells shown as enlarged images on right side. F) Reduced cell migration and invasion ability in SMC‐treated Hep3B cell visualized and quantified (lower panel) (*n* = 3). G) In vivo tumorigenicity test: tumor formation in nude mice engrafted with SMC‐treated and control Hep3B cells. Left dorsal flanks: control cells; right dorsal flanks: SMC‐treated cells (*n* = 4). Hep3B and HepG2: liver cancer cell lines. T2W and T4W represent SMC treatment for 2–4 weeks. Scale bar, 100 µm. Data represent the means ± SD. Asterisks indicate significance compared to control untreated cells as assessed by *t*‐test: *p < 0.05; **p < 0.01; ***p < 0.001.

We first applied SMC on liver cancer cell line HepG2 and Hep3B. After 14–28 days of SMC treatment, we observed consistent loss of malignant features in treated cells, including growth arrest, increased senescent G0/G1 population, reduced EdU incorporation rate (**Figure** **1**A–C), increased cell apoptosis (Figure 1D), as well as diminished colony formation and cell migration and invasion ability (Figure 1E,F). Elimination of malignant characteristics attested to the change of cell identity. For validation, we performed the same experiments using other liver cancer cell lines such as CSQT‐2, a cell line with strong metastatic and invasive abilities, and BEL‐7402/5Fu, which is resistant to chemotherapy agent 5Fu, and received similar results (Figure S2A–F, Supporting Information).

Next, we tested the in vivo tumorigenicity potential of the SMC‐treated Hep3B cells by injecting them and their control counterparts into nude mice. 4 weeks post cell injection, we observed tumor emergence in the left flank of mice where Hep3B cells were injected, whereas the right side, which received injection of SMC‐treated Hep3B cells, showed no sign of tumor formation (Figure 1G; Figure S2G, Supporting Information). We conducted a similar inoculation experiment by injecting double amount of treated BEL‐7402/5‐Fu cells (2 × 10^6^/point) into both flanks of mice, and extended observation window till 24 weeks, and still found no sign of tumor development (data not shown). Together, these results demonstrated that SMC treatment eliminated malignancy of different liver cancer cells, including a drug‐resistant subtype.

### 2.3 SMC‐Treated Liver Cancer Cells Regain Hepatocyte Phenotype

**Figure 2:**
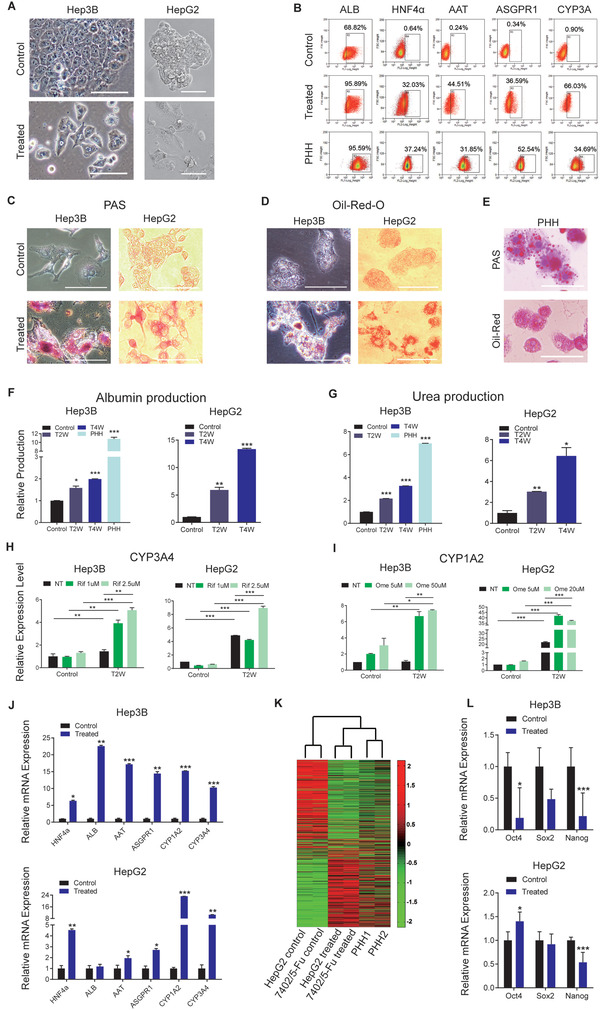
SMC‐treated liver cancer cells regain hepatic cell features and functions. A) Representative images of SMC‐treated and control cells on day 20 of culturing. B) Expression of hepatocyte‐specific markers measured by FACS analysis. Primary human hepatocytes (PHH) were used as positive control. Cytoplasmic accumulations of glycogen and lipid were determined by C) PAS and D) oil‐red O staining. E) Primary human hepatocytes (PHH) were used as positive control. F) Relative ALB production and G) urea synthesis of 20 days SMC‐treated cells (*n* = 3). Induction of CYP genes in 20 days SMC‐treated and control cells. H) CYP3A4 and I) CYP1A2 were induced by the indicated concentrations of rifampicin and omeprazole, respectively, for 48 hours (*n* = 3). Rif, rifampicin; Ome, omeprazole. J) Expression of hepatocyte‐specific genes in 20 days SMC treated or control samples (*n* = 3). K) Comparison of gene expression profiles of SMC‐treated (20 days) and control cells. SMC‐treated samples could be clustered with PHH by a Pearson correlation analysis. L) mRNA levels of pluripotency genes measured by qRT‐PCR assays (*n* = 3). Scale bar, 100 µm.

In addition to losing their malignant characteristics, liver cancer cells treated by SMC reacquired normal flat phenotype of fully differentiated somatic cells, with clear margins and nuclei, as well as scattered distribution in contrast to their control counterparts’ blurry appearance and colony growth pattern (**Figure** **2**A). In the meantime, expression of hepatocyte‐specific markers was also observed in SMC‐treated samples (Figure 2B; Figure S3A, Supporting Information).

SMC‐treated cells also gained key hepatocyte functions such as glycogen storage (Figure 2C), lipid metabolism (Figure 2D; Figure S3B, Supporting Information), albumin secretion and urea synthesis (Figure 2F,G; Figure S3C,D, Supporting Information). P450 cytochrome activities, including CYP3A4 and CYP1A2, could also be induced under rifampicin and omeprazole induction, respectively (Figure 2H,I; Figure S3E, Supporting Information), suggesting restored drug metabolizing ability in the SMC‐treated cells.

In addition, hepatic genes had markedly increased expressions in SMC‐treated samples (Figure 2J). Genome‐wide expression profile of SMC‐treated HepG2 and 7402/5‐Fu cells exhibited opposite patterns to those of their control counterparts: genes that strongly expressed in control cells were suppressed in SMC‐treated samples (Figure 2K). Moreover, the expression patterns of treated cells clustered with those of primary hepatocytes (Figure 2K) (PHH data from GEO, accession number GSE42643^[^ ^13^ ^]^) suggesting reestablishment of hepatic cell identity. Pluripotency genes, such as Oct4, Sox2 and Nanog (Figure 2L), were however down‐regulated in SMC‐treated samples, indicating that the differentiated liver cancer cells did not possess stemness properties.

Together, these data indicated that SMC successfully induced differentiation of multiple types of liver cancer cells. It not only deprived their malignant features, but also restored phenotype of hepatocytes, including morphology, marker expressions, functions and gene expressions.

### 2.4 SMC Consistently Induces Liver Cancer Cell Differentiation In Vitro Regardless of Heterogeneity

**Figure 3:**
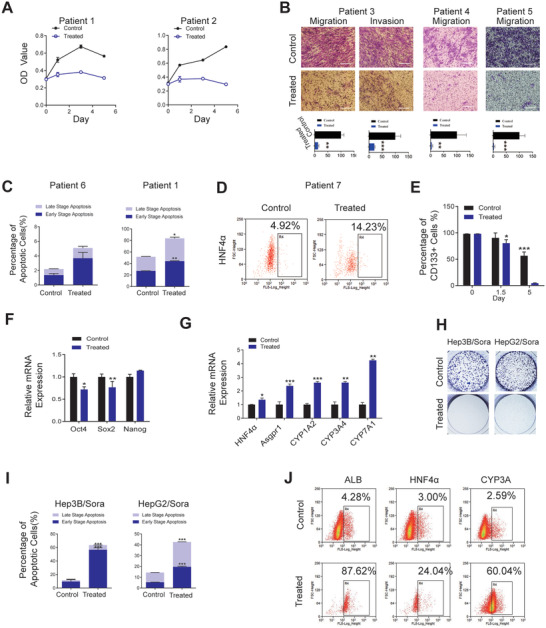
SMC consistently induce differentiation of patient‐derived primary cancer cells, cancer stem cells (CSCs) and drug‐resistant cancer cells. A) Relative cell viability of control and SMC‐treated patient‐derived primary cancer cells (*n* = 3). B) Reduced cell migration and invasion ability of SMC‐treated cells visualized and quantified (lower panel) (*n* = 3). Scale bar, 50 µm. C) FACS analysis of apoptotic populations after SMC treatment for 3 days using annexin V staining (*n* = 3). D) HNF4a expression on day 7 SMC‐treated cells by FACS analysis (*n* = 3). E) CD133^+^ population in HCC cell Huh7 treated with SMC for 1.5 and 5 days measured by FACS analysis (*n* = 3). F) Expression of pluripotent genes in CSCs treated with SMC for 14 days (*n* = 3). G) Hepatocyte‐specific gene expressions in CSCs treated with SMC for 14 days (*n* = 3). H) Loss of cell colony formation ability in SMC‐treated sorafenib‐resistant cells visualized. I) FACS analysis of apoptotic sorafenib‐resistant populations after SMC treatment for 14 days using annexin V staining (*n* = 3). J) Expression of hepatic markers in sorafenib‐resistant HepG2 cells treated by SMC for 14 days measured by FACS analysis. Scale bar, 100 µm.

After we demonstrated that SMC could induce differentiation of different liver cancer cell lines, we further explored whether heterogeneous backgrounds of liver cancer cells could affect the potency of SMC. We first applied this cocktail on primary liver cancer cells derived from 9 Asian liver cancer patients, including 6 males and 3 females between the ages of 40–67. Among these patients, 6 had liver cancers caused by hepatitis B virus (HBV)/hepatitis C virus (HCV)‐induced liver cirrhosis; and 1 patient already developed lymph node metastasis. Gene expression profiles of these patients’ cell samples (4/7) also attested to their heterogeneity (Figure S4, Supporting Information). Under SMC treatment, we observed consistent loss of malignant features in all primary liver cancer cell samples, including arrested cell proliferation, diminished cell migration, and invasion ability, as well as partial cell apoptosis (**Figure** **3**A–C; Figure S5A–D, Supporting Information). The remaining cells, however, showed elevated HNF4*α* expression, suggesting differentiation (Figure 3D; Figure S5E,F, Supporting Information).

Cancer stem cell (CSCs) is a major contributor to cancer recurrence, metastasis, and drug resistance.^[^ ^14^, ^15^, ^16^ ^]^ In the meantime, their stemness properties also makes them ideal targets for the differentiation strategy. We screened for CSCs in multiple liver cancer cell lines based on previously established stemness markers^[^ ^17^ ^]^ and eventually identified a subclone of the liver cancer cell line Huh7 expressing CD133 as the test subject (Figure S6, Supporting Information). By FACS sorting, we enriched CD133‐positive cancer stem cells and treated them with SMC. FACS analysis showed that, as early as day 5, the CD133+ cell population in SMC‐treated sample already decreased to less than 5%, while the control sample still had approximately 60% (Figure 3E). qRT‐PCR result revealed that the expression of stemness genes such as Oct4 and Sox2 were significantly decreased (Figure 3F), meanwhile the expressions of hepatic genes were increased (Figure 3G) indicating elimination of CSCs through SMC‐induced differentiation.

We then sought to determine whether the differentiation strategy is effective on cancer cells resistant to targeted therapy, which represents a major hurdle for current treatment paradigm. We used Hep 3B and HepG2 as parental cell lines to establish two Sorafenib‐resistant subclones Hep 3B/Sora and HepG2/Sora (Figure S7), and subjected them to SMC treatment for 21–28 days. The changes occurred on both cell lines were similar to those occurred on their parental cell lines, including lost colony formation ability, increased cell apoptosis and increased hepatic marker expression (Figure 3H–J), indicating that SMC could induce differentiation of drug‐resistant cancer cells as well.

Combined with previous results on different cancer cell lines, our studies suggested that SMC‐induced differentiation strategy is generally applicable to liver cancer cells of various origins, backgrounds, and features. Not only can it revert the phenotype of average liver cancer cells, but it can also eliminate tumor‐initiating cancer stem cells and drug‐resistant cancer cells.

### 2.5 SMC Induces Liver Cancer Cell Differentiation on CDX and PDX Models In Vivo

**Figure 4:**
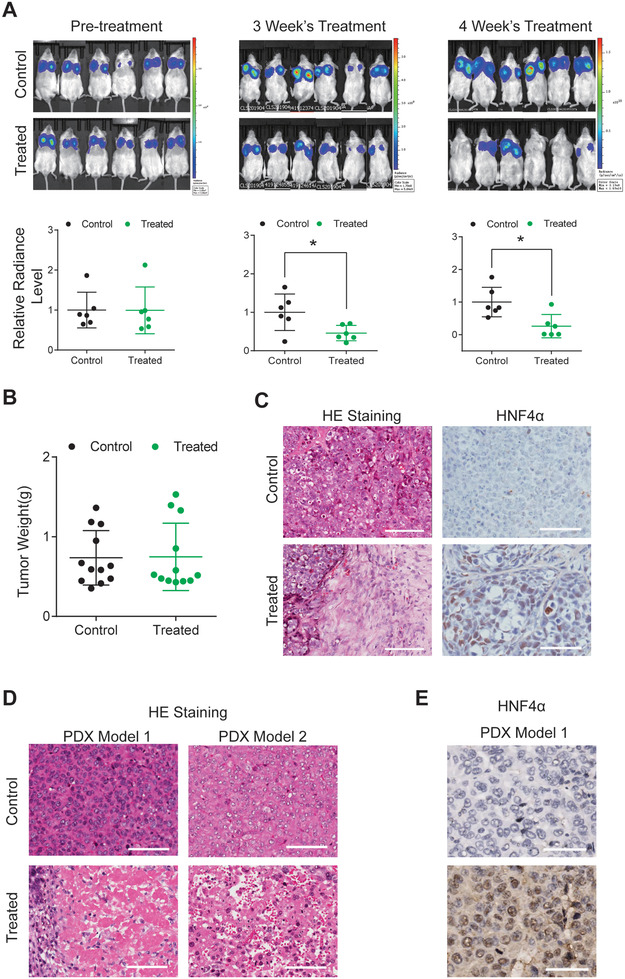
Differentiation strategy exhibits anti‐tumor ability on CDX and PDX animal models. A) Radiance intensity of luciferase‐labeled Hep3B cells on CDX mice model treated with SMC or control condition measured by in vivo imaging. Upper lanes: IVIS image, lower lanes: Statistic analysis (*n* = 12, 6 mice, 12 tumors). B) Statistic analysis of tumor weights after 4 weeks of SMC or control treatment (*n* = 12). C) Representative photomicrographs of HE staining showing tissue necrosis in tumors dissected from Hep3B CDX models treated with SMC. The remaining tumor tissue had positive HNF4*α* staining (*n* = 12). D) Representative photomicrographs of HE staining showing tissue necrosis in tumors dissected from liver tumor PDX mice models treated with SMC for 4 weeks. E) The remaining tumor tissue had positive HNF4*α* staining (*n* = 12). Scale bar, 100 µm.

We then examined the impact of chemical‐induced differentiation strategy on liver cancer in vivo. mCherry‐luciferase labeled Hep3B cells were transplanted subcutaneously into both dorsal flanks of nude mice to establish a cell‐derived xenograft (CDX) model. Upon SMC treatment through daily intra‐tumor injection, the luciferase activities measured by in vivo imaging were substantially reduced in treated animals (**Figure** **4**A; Figure S8A, Supporting Information), suggesting diminished tumor cell viability. Though we found no significant difference in terms of volume and weight between tumors of the control and treated animals (Figure 4B; Figure S8B, Supporting Information), pathological analysis of dissected tumors revealed wide range of tissue necrosis and blood cell infiltration within tumors of the SMC‐treated animals, confirming previous luciferase imaging data (Figure 4C; Figure S8C, Supporting Information, left panel). Remaining tumor tissues, which were mainly located on tumor rims next to normal tissues, were found to highly express the hepatic nuclear marker HNF4*α*, suggesting restoration of hepatocyte's identity (Figure 4C; Figure S8C, Supporting Information, right panel).

To further confirm the strategy's potency and explore whether it would be impacted by cancer heterogeneity in vivo, we constructed patient‐derived xenograft (PDX) animal models through subcutaneous implantation (6 mice, 2 tumors/mice). After 4 weeks of treatment by SMC, dissection of tumors revealed similar histological results to those from CDX models: extensive tissue necrosis accompanied by large amount of blood cell infiltration within the tumors (Figure 4D), and positive HNF4*α* expression in the remaining tumor tissues (Figure 4E), indicating reversion to normal phenotype.

### 2.6 The Strategy Exhibits Anti‐Cancer Effect on Orthotopic CDX, PDOX As Well As Metastatic Liver Cancer Models

**Figure 5:**
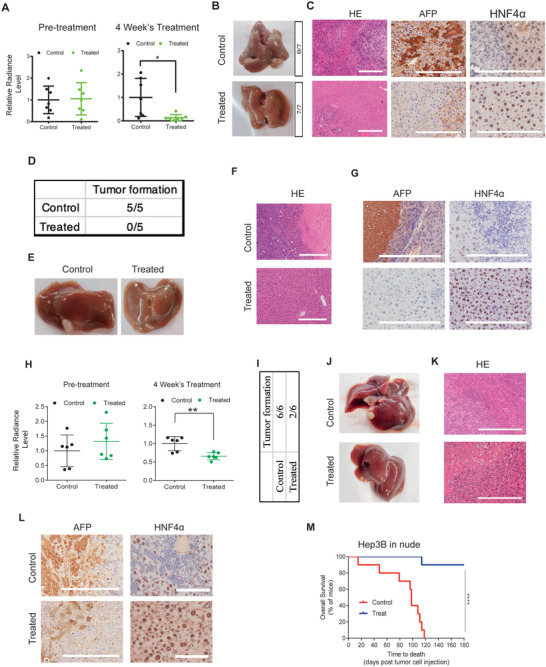
The anti‐tumor effect of SMC on orthotopic CDX, PDOX, and metastatic liver tumor models. A) Radiance intensity of luciferase‐labeled Hep3B cells on orthotopic CDX mice model treated with SMC or control measured by in vivo imaging (*n* = 7). B) Representative images of livers dissected from control and SMC‐treated orthotopic CDX mice and the number of tumors observed (right panel). C) Representative photomicrographs of HE staining, AFP and HNF4*α* immunohistochemical staining showing tissue necrosis in liver dissected from SMC‐treated mouse model (HE, left panel). Few cells with weak AFP staining in the SMC treated samples in contrast to large area of AFP positive staining in the control samples (middle panel). Enhanced HNF4*α* staining in treated samples (right panel) (*n* = 7). D) The number of tumors emerged in livers of PDOX mouse models (*n* = 5). E) Representative images of livers dissected from control and SMC‐treated orthotopic PDOX mice (*n* = 5). F) Representative photomicrographs of HE staining showing tumor tissue dissected from control and SMC‐treated PDOX models (*n* = 5). G) AFP and HNF4*α* immunohistochemical staining of control and SMC treated PDOX models’ liver tissue samples (*n* = 5). H) Radiance intensity of luciferase‐labeled liver cancer cells on metastatic CDX mice model treated with SMC or control measured by in vivo imaging (*n* = 6). I) Number of tumors observed on livers. J) Representative images of livers dissected from control and SMC‐treated metastatic mice models (*n* = 6). K) Representative photomicrographs of HE staining showing tumor tissue dissected from control and SMC‐treated metastatic mice models. Necrosis tissue could be observed in SMC treated samples (*n* = 6). L) AFP and HNF4*α* immunohistochemical staining of control and SMC treated liver tissue samples (*n* = 6). M) Kaplan–Meier survival curve of SMC treated and control metastatic CDX models (*n* = 9). Scale bar, 100 µm.

Despite the wide application of subcutaneous xenograft models in anti‐tumor research, there is still criticism in regards to its inability to mimic tumor's microenvironment, which is often critical to disease progression. In order to better test the strategy's efficacy and further increase clinical translatability, we constructed an orthotopic CDX mouse model by injecting 1 × 10^6^ mCherry‐luciferase labeled Hep3B cells into mouse liver, and waited 3 weeks to allow tumor formation. Animals were treated with SMC or saline solution through oral administration for 4 weeks. We found that SMC treatment led to significant reduction in tumor cell viability as measured by luciferase activity (**Figure** **5**A). Accordingly, tissue dissection at the end of experiment showed emergence of tumor nodules in the livers of control animals (7/7), but no observable tumor existed in SMC‐treated animals’ livers (0/7) (Figure 5B,C, left panel). The results were supported by alpha‐fetoprotein (AFP) immunohistochemical staining on liver sections. Large AFP positive areas were observed in control animals’ liver tissue sections, while no or only a few weak positive cells were found in liver sections of SMC‐treated mice (Figure 5C middle panel). In contrast, SMC‐treated animals’ liver tissues widely stained positive for HNF4*α*, suggesting SMC induced differentiation (Figure 5C right panel).

In order to ensure that the heterogeneity aspect was also captured in our orthotopic liver cancer animal models, we constructed patient‐derived orthotopic xenograft (PDOX) models by implanting patient‐derived xenografts into hepatic subcapsular regions of mice, and waited 4 weeks to allow tumor formation. Animals were then randomly divided into 2 groups and treated with control solution or SMC for 4 weeks (5 mice/group). At the end of experiment, through tissue dissection, we found that tumors emerged in the liver of every animal in the control group (Figure 5D–F), some on the surface (2/5) while others embedded in liver tissues (3/5) (Figure 5E). However, no sign of tumor formation was observed in the SMC‐treated animals (Figure 5D–F). Consistent with this observation, immunohistochemical staining on liver sections showed that large number of AFP‐positive tumor cells existed in the control animals’ liver tissue samples, but not in SMC‐treated samples (Figure 5G, left panel). In contrast, extensive HNF4*α*‐positive cells were observed in SMC‐treated animals’ liver tissues, but only very few HNF4*α*‐positive cells could be spotted in the control samples (Figure 5G right panel). Given that previous study already reported that overexpression of HNF4*α* would lead to differentiation of liver cancer cells, the staining results indicated that SMC effectively abrogated liver tumors by inducing cancer cell differentiation in vivo.

A common subtype of liver cancer is metastatic hepatocellular carcinoma (HCC), which presents a major hurdle to therapeutics due to its unusual invasiveness. To test the efficacy of the differentiation strategy against this type of cancer, we generated a metastatic HCC model through spleen injection of 1 × 10^6^ mCherry‐Luciferase labeled HCC cells. After waiting 3 weeks for tumor formation, SMC was then applied through oral administration for 4 weeks. The level of luciferase activity indicated that SMC treatment led to reduced tumor cell viability (Figure 5H). Even though we still found emergence of tiny tumors in the livers of some SMC‐treated animals, the differentiation strategy significantly reduced the number and volume of the tumor nodules in treated animals (Figure 5I,J). Consistently, notably decreased expression of AFP was observed in liver tissue sections of SMC‐treated animals in comparison to those of control animals (Figure 5L), while expression of HNF4*α* significantly increased, suggesting differentiation of HCC cells (Figure 5L, right panel). Consequently, life spans of mice were significantly extended in the treated group (Figure 5M).

### 2.7 SMC Shows Anti‐Tumor Effect on Drug‐Resistant Liver Tumor Animal Models

**Figure 6:**
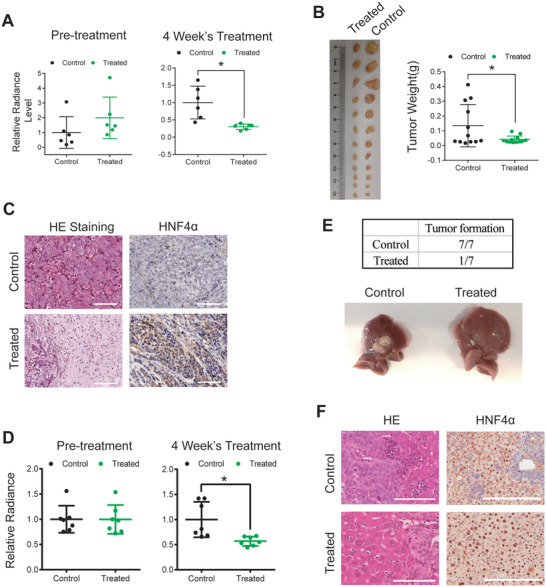
The anti‐tumor effect of SMC on drug‐resistant CDX Model. A) Radiance intensity of luciferase‐labeled Hep3B/Sora cells on subcutaneous CDX mice models before and after SMC treatment (*n* = 12, 6 mice, 12 tumors). B) Images and weights of tumors dissected from subcutaneous Hep3B/sora CDX mice models treated with control or SMC. Left panel: dissected tumor images (*n* = 12). C) Representative photomicrographs of HE staining and HNF4*α* immunohistochemical staining showing tissue fibrosis in tumors dissected from subcutaneous Hep3B/sora CDX mice models treated with SMC. Remaining tumor tissue had positive HNF4*α* staining (*n* = 12). D) Radiance intensity of luciferase‐labeled Hep3B/Sora cells on metastatic CDX mice models before and after SMC treatment (*n* = 7). E) Number of tumors observed on animal livers (upper table) and the representative images of livers dissected from control and SMC‐treated metastatic Hep/Sora CDX mouse models (*n* = 7). F) Representative photomicrographs of HE staining and HNF4*α* immunohistochemical staining showing liver tissues dissected from metastatic Hep3B/sora CDX mice models treated with SMC or control (*n* = 7). Scale bar, 100 µm.

Cancer heterogeneity is also reflected by different responsiveness to drugs. To test the efficacy of chemical‐induced differentiation on drug‐resistant tumors, we reconstructed previous subcutaneous CDX model using Hep3B/Sora cells (Sorafenib‐resistant Hep3B cells) labeled with mCherry‐Luciferase. We injected Hep3B/Sora cells subcutaneously into both dorsal flanks of nude mice, and waited 2 weeks to allow tumor formation. Then, animals were treated with SMC or control condition through intra‐tumor injection for 4 weeks. Once again, we observed substantial reduction of tumor cell viability in SMC treated animals by monitoring luciferase activity (**Figure** **6**A). Unlike previous Hep3B cell‐derived CDX models, where no difference of tumor weights was observed in control and SMC treated animals, tumor burdens were remarkably reduced in SMC treated group in this experiment (Figure 6B). In addition, SMC treatment did not induce necrosis of tumor tissues, but rather wide spread fibrosis, as revealed by dissection of the tumors (Figure 6C, left panel). Nevertheless, remaining tissues on tumor rims still expressed HNF4*α* (Figure 6C, right panel).

We also reconstructed previous metastatic HCC model using drug‐resistant Hep3B/Sora cells (Figure 6D). After 4 weeks of SMC treatment, similar results were obtained including reduced tumor cell viability as measured by luciferase activity (Figure 6D), and reduction in tumor nodule's size and number (Figure 6E). Increased HNF4*α* expression in liver tissues attested liver cancer cell differentiation induced by SMC treatment (Figure 6F).

Taken together, these in vivo data not only further confirmed the anti‐tumor potency of the chemical‐induced differentiation strategy, but they also proved that the efficacy of this strategy would not be affected by cancer cell heterogeneity, including features such as drug‐resistance.

### 2.8 SMC‐Induced Suppression of PI3K/Akt/mTOR and Snail Signaling Contributes to Differentiation of Liver Cancer Cells

**Figure 7:**
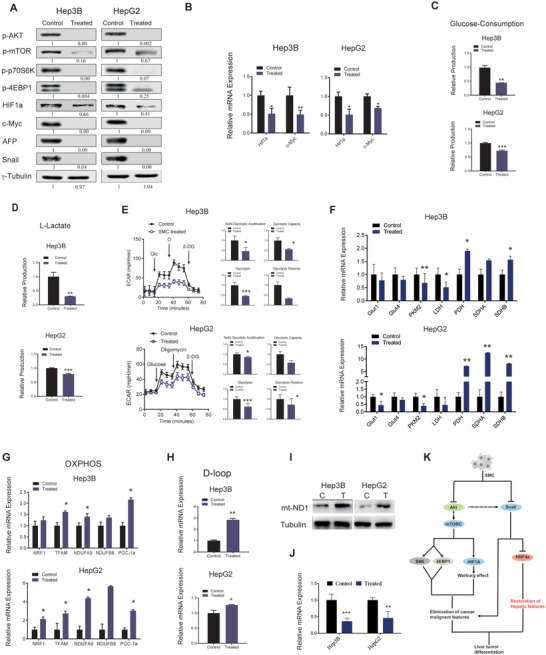
Down‐regulation of PI3K/AKT/mTOR signaling and Snail activity as well as reprogramming metabolic pattern by SMC treatment contribute to the liver cancer differentiation induction. A) Protein levels of SMC‐treated liver cancer cells on day 8 of culturing determined by Western‐blot assay and relative quantification. B) Expression of HIF1a and c‐Myc genes in SMC‐treated cell samples on day 8 (*n* = 3). C) Relative glucose consumption and D) lactate production of SMC‐treated cell samples on day 7 (*n* = 3). E) Extracellular acid ratio (ECAR) of SMC‐treated liver cancer cells on day 7 measured by XF96 Extracellular Flux Analyzer. 2‐DG, 2‐deoxyglucose (*n* = 3). F) Expression of key glucose metabolism enzyme genes on SMC‐treated cells on day 8 (*n* = 3). G) Expression of OXPHOS‐related genes in control and SMC‐treated cells on day 8 of treatment (*n* = 3). H) Gene expression of mitochondrial D‐loop in treated cells on day 8 (*n* = 3). I) mtDNA‐encoded subunits ND1 expression. J) Snail gene expression in SMC‐treated liver cancer cells after 4 days. K) Mechanism scheme of liver cancer cell differentiation induction by SMC.

PI3K/Akt/mTOR pathway is the most commonly activated signaling in human cancers and plays critical role in tumor cell survival, growth, angiogenesis, migration, and invasion, as well as metabolism regulation.^[^ ^18^ ^]^ Through augmenting the activity of nutrient transporters and metabolic enzymes, PI3K/Akt pathway can reprogram cancer cellular metabolism to support the anabolic demands of aberrantly growing cells. To examine the PI3K/Akt/mTOR signaling activity under SMC treatment, we performed western blot assay, and found that SMC led to down‐regulated phosphorylation levels of Akt, mTOR and the major targets of mTOR complex 1: S6K and 4E‐BP1 proteins (**Figure** **7**A), which are two key factors involved in protein synthesis. The direct downstream targets of mTOR—HIF1*α* and c‐Myc that hold crucial roles in regulating cell metabolism by targeting glycolytic enzymes^[^ ^19^ ^]^ were also inhibited under SMC treatment, at both protein and mRNA levels (Figure 7A,B).

The Warburg effect, which refers to reprogrammed cancer cell metabolism that favors aerobic glycolysis over oxidative phosphorylation (OXPHOS),^[^ ^20^ ^]^ is a core feature of liver tumor development. Suppression of the Warburg effect served as the driving force that directed differentiation of glioblastoma cells into normal astrocytes.^[^ ^21^ ^]^ To test whether SMC treatment affected liver cancer cells’ metabolic pattern, we detected glucose consumption and lactate production of SMC‐treated liver cancer cell samples, and found both to be significantly decreased (Figure 7C,D). We then measured cell extracellular acid ratio (ECAR) using the XF96 extracellular flux analyzer, which is a marker of glycolytic abilities of cells. As expected, ECAR was strongly suppressed in treated liver cancer cells as well (Figure 7E). We also measured cell oxygen consumption ratio (OCR) and found that it was enhanced (Figure S9, Supporting Information), indicating that SMC treatment reprogrammed the liver cancer cell's metabolic pattern from aerobic glycolysis to OXPHOS.

Next, we detected the gene expression of glycolysis pathway key enzymes such as GLUT1, GLUT4, PFKFB3, PKM2, and LDH, etc., and found that these key glycolysis enzymes (GLUT1, GLUT4, PFKFB3, and LDH) were significantly decreased in SMC treated samples (Figure 7F). Conversely, expression of OXPHOS‐related factors was strongly enhanced (Figure 7G). Apart from these, the number of mitochondria and expression of mtDNA‐encoded subunits ND1 were also significantly increased (Figure 7H,I), indicating elevated mitochondrial activity.

Next, we explored which possible node prompted cell's acquisition of hepatocyte characteristics. Snail plays a central role in epithelial‐to‐mesenchymal transition (EMT) signaling, with the ability to trigger EMT cascade. Snail can directly bind to HNF4*α* promoter and repress the transcription of HNF4*α* gene, consequently affect hepatocyte epithelial morphogenesis and differentiation.^[^ ^22^, ^23^ ^]^ Through western blot assay, we found that SMC treatment strikingly down‐regulated the expression of Snail (Figure 7A). Snail gene expression was also significant reduced (Figure 7J). This observation, combined with our previous results showing that HNF4*α* had elevated expression both in vitro and in vivo, suggesting that SMC‐induced Snail suppression may contribute to the liver tumor cell fate conversion and their acquisition of hepatocyte characteristics.

Taken together, through suppression of PI3K/Akt signaling and Warburg effect, SMC treatment led to elimination of tumor malignant features (Figure 7K). The Snail1 suppression resulted in HNF4*α* activation which promoted the restoration of hepatic features in remaining living liver cancer cells (Figure 7K). Additionally, Snail is also involved in the loss of tumor malignant features (Figure 7K).

## 3 Discussion

Differentiation therapy has not made many progresses as a cancer treatment avenue since the hallmark success of treating APL with ATRA.^[^ ^4^, ^6^, ^10^ ^]^ It was difficult to replicate the rapid clearance of APL cells under ATRA induction, particularly on solid tumors, since most of them possess far more complex genetic basis than leukemia. Among solid tumors, liver cancer in particular has high level of inter‐ and intra‐tumor heterogeneity.^[^ ^9^ ^]^ It lacks a dominant oncogene driver, which greatly hampered drug development efforts. Inspired by developments in the field of stem cells and cell reprogramming studies,^[^ ^24^, ^25^, ^26^, ^27^ ^]^ we theorized that the right combination of chemicals could solve the challenge of solid tumor differentiation. In this study, we demonstrated that a selected small molecule cocktail (SMC) could indeed induce differentiation of liver cancer cells, and consequently eliminate their malignant features and restore hepatocyte‐like identity in vitro. When administered in vivo, the SMC strongly suppressed the progression of tumors. More importantly, the genetic variances between different liver cancer cells (Figure S4, Supporting Information) and the unique features such as stemness or drug resistance, had little impact on this strategy's potency both in vitro and in vivo. The SMC displayed consistent anti‐tumor efficacy on subcutaneous, orthotopic and metastatic cell‐derived or patient‐derived xenograft mice models, which makes it an ideal approach against liver cancer, with translational potential.

Our results indicate that SMC likely induced differentiation of liver cancer cells by working on two aspects through two key nodes. The first aspect is the elimination of cancer cell malignant features through SMC induced inhibition of PI3K/Akt/mTOR signaling pathway axis (Figure 7K). It is well‐known that PI3K/Akt/mTOR pathway play essential role in cellular activities.^[^ ^28^ ^]^ It is also abnormally activated in many types of cancers including liver tumor.^[^ ^29^ ^]^ Inhibition of this signaling directly leads to suppressed protein synthesis and aerobic glycolysis activities in cancer cells. Aerobic glycolysis, as a hallmark of cancer, is essential for malignant cell survival, growth, angiogenesis, invasion, and drug‐resistance. Alterations in metabolism pattern can also in turn affect cellular signaling, epigenetics, and gene expression. In our case, SMC treatment reprogrammed liver cancer cells’ metabolic pattern through modulating the PI3K/Akt/mTOR axis. As a result, aerobic glycolysis was significantly decreased while OXPHOS and mitochondrial activity were enhanced. The inhibition of protein synthesis, in combination with the suppression of aerobic glycolysis, eventually led to eradication of liver cancer cell's malignancy.

Other than the loss of tumor cell features, another important part of liver cancer cell differentiation is the acquisition of hepatocyte characteristics for the cells that did not end in apoptosis. EMT is a core process involved in tumorigenesis of epithelial cells.^[^ ^30^, ^31^, ^32^ ^]^ Snail, as the central factor of EMT, is regulated by Akt, HIF1*α*, TGF*β* signaling, etc. In hepatocytes, previous researches have shown that Snail could directly bind to HNF4*α* promoter and repress its gene transcription, consequently affect hepatocytes epithelial morphogenesis and differentiation state.^[^ ^22^, ^23^ ^]^ The role of HNF4*α* in hepatocyte differentiation, proliferation, and long term phenotype maintenance is well recognized and extensively studied.^[^ ^33^ ^]^ Ectopic expression of the HNF4*α* could also induce differentiation of liver cancer cells.^[^ ^11^, ^34^ ^]^ In our study, we found that SMC treatment resulted in strikingly suppression of Snail, which not only can inhibit the EMT process and support the malignancy eradication, but can also enhance HNF4*α* expression, thus allow SMC‐treated liver cancer cells to reacquire hepatocyte features and identity. This was confirmed in our experiments both in vitro and in vivo, where we observed elevated HNF4*α* expression in treated animals’ liver samples.

It is worth noting that even though SMC's induction can lead to metabolic changes and eventual differentiation of liver cancer cells, we did not find it incurring any negative effect on primary hepatocytes (Figures S10 and S11, Supporting Information). Hepatocytes did not seem to suffer from apoptosis, senescence, or inhibited proliferation (Figures S10 and S11, Supporting Information). In fact, the phenotype of primary hepatocytes cultured with SMC was better maintained than cells cultured in normal conditions. This phenomenon was, however, reasonable, as some components of SMC overlapped with chemicals for long‐term culturing of primary hepatocytes in vitro.^[^ ^35^ ^]^ We also did not find SMC suppressing liver regeneration in 2/3 partial hepatectomy (Figure S12, Supporting Information) indicating that SMC has little impact on the original liver's abilities. Even though previously some researches have demonstrated that high HNF4*α* expression level could inhibit cell proliferation, we did not observe such effect in our research either in vitro or in vivo. The reason is, perhaps, that SMC treatment only led to slight elevation of HNF4*α* expression in normal primary hepatocytes, whose expression level is already fairly high, unlike in liver cancer cells, where SMC's induction could enhance the HNF4*α* expression much more significantly. This cascade, on one hand, allows restoration of the hepatic features in liver cancer cells, on the other hand, may also help inhibiting proliferation of malignant cells.

Despite these highly encouraging results, there are several questions that need to be addressed prior to translational studies. First of all, how the four chemicals function synergistically to overcome tumor heterogeneity and induce differentiation of various liver cancer cells? Our study revealed that cell signaling and metabolic modulation played important roles, but this may only be part of the puzzle. More investigations on other aspects such as cell epigenetic are still required to thoroughly understand the cell fate transition mechanisms. In addition, the optimal ratio of components and dosage of the combination requires further, in‐depth investigation. Analysis of pharmacokinetics and pharmacodynamics should also be conducted on the SMC to assess potential drug‐drug interactions. Finally, even though the SMC‐treated animals under our observation showed no decrease in body weight, more rigorous exploration is needed to evaluate long term toxicity of SMC. Given that it is a four‐factor combination, if some of SMC's components can be replaced by marketed drugs with clear safety profile, then this approach would have a better opportunity to be translated into a real therapeutic option.

## 4 Conclusion

Regardless, as our work indicates, chemical‐induced differentiation therapy not only may serve as a highly effective approach against liver cancer, but may also shed some lights on the core issue of cancer heterogeneity as a whole.

## 5 Experimental Section

### Small Molecules

Please refer to Table S1, Supporting Information, for details.

### Cell Culture

Liver cancer cell lines were purchased from ATCC and Cell Bank of Chinese Academy of Sciences (Shanghai, China). For more detailed information please refer to Supporting Information.

### Sorafenib Resistant Cell Line Establishment

Sorafenib resistant cell lines (Hep3B/Sora and HepG2/Sora) were established through stepwise selection of surviving Hep3B and HepG2 cells that were exposed to increasing doses of sorafenib (Sora) from 1 µm till final dosage of 15 µm.

### Cell Apoptosis Detection

Cell apoptosis detection was performed according to the manufacturer's instructions (Dead Cell Apoptosis Kit, Invitrogen, Catalog No. V13245). For more detailed information please refer to Supporting Information.

### Cell Growth Ability Evaluation

Cell proliferation assay was carried out using Cell Counting Kit‐8 (CCK‐8; Dojindo Laboratories). For more detailed information please refer to Supporting Information.

### Cell Cycle Detection

Cell cycle histograms were determined using PI staining by flow cytometry. For more detailed information please refer to Supporting Information.

### Edu Incorporation Assay

Multiple procedures were carried out step by step according to the manufacturer's instructions. For more detailed information please refer to Supporting Information.

### PAS Staining, Oil Red O Staining

The PAS and Oil Red O staining kits were purchased from Njjcbio. Cells were fixed with 4% paraformaldehyde in PBS and stained according to the manufacturer's instructions.

### Immunofluorescent Staining

Cells were blocked with PBS containing 0.2% Triton X‐100 and 5% normal goat serum (NGS) and then incubated with primary antibodies. After incubated with appropriate secondary antibodies, cells were washed again and analyzed by FACS. For detailed information please refer to Supporting Information.

### The Quantitative of Albumin and Urea Production

Human albumin and urea assay kits were purchased from BioAssay Systems, albumin and urea detection were performed according to the manufacturer's instructions. An extra well of total cell DNA content was used to normalize the data. For detailed information please refer to Supporting Information.

### The Induction of CYP Metabolism Activity

To induce CYP metabolism activities, cells were cultured in 12‐well plate for 24 h and then replaced medium supplemented with chemical inducers (omeprazole for CYP1A2, rifampicin for CYP3A4) daily for 48 h, then total RNA was isolated and CYP gene expression level was quantitative using real‐time RT‐PCR assay.

### Plate Colony Formation Assays

Control and SMC‐treated cells were trypsinization and counting, 1000 live cells were seeded in 6‐well plates in triplicate, and cultured in 2–2.5 mL medium for 10–15 days. Cells were fixed and performed crystal violet staining.

### Migration and Invasion Assay

Control and SMC‐treated cells were trypsinization and counting. Live cells were seeded on polycarbonate transwell membrane to reach almost 100% confluent (Greiner Bio‐One) which match 24‐well plate. Cells were fixed and stained after a certain period according to the variations of different cells’ migration ability.

### Primary Liver Cancer Cells Isolation and Culture

Tumor tissue that derived from liver cancer were digested using collagenase type IV solution in 37 °C for 20–30 min. Fresh isolated tumor cells were cultured in DMEM with 10% FBS, 1% ITS supplement, 20 ng mL^−1^ EGF addition. Medium were changed every two days. For more detailed information please refer to Supporting Information. All experiments involving use of human tissue samples were approved by the Research Ethics Committee of Eastern Hepatobiliary Surgery Institute (approval number EHBHKY2015‐K‐001)

### Xenograft Studies

Mice were manipulated and housed according to the criteria outlined in the “Guide for the Care and Use of Laboratory Animals” prepared by the Eastern Hepatobiliary Surgery Institute. All xenograft experiments were approved by the Research Ethics Committee of Eastern Hepatobiliary Surgery Institute (approval number EHBH‐DWLL‐023).

### In Vivo Tumor Formation Experiment

For more detailed information please refer to Supporting Information.

### Cell‐Derived Xenograft (CDX), Patient‐Derived Xenograft (PDX), and Metastatic CDX Mouse Models Generation and Treatments

6–8 weeks old BALB/C male nude mice were purchased from Sino‐British SIPPR/BK Lab Animal Ltd. For more detailed information please refer to Supporting Information.

### Quantitative RT–PCR

Total RNA was isolated from cells by TRIzol (Invitrogen) in the standard protocol, and reverse transcription was performed using M‐MLV Reverse Transcriptase (Invitrogen) by using 2 µg RNA. Quantitative PCR was performed using a Roche Light Cycler 96 System (Roche, USA) and Sybr Green Supermix (Bio‐Rad Laboratories). For more detailed information please refer to Supporting Information.

### SDS–PAGE and Western Blot Analysis

Whole‐cell lysates were run on 10% SDS‐polyacrylamide gels and transferred to nitrocellulose membrane (GE) by standard methods. For more detailed information please refer to Supporting Information.

### Mitochondrial Stress Test and Glycolysis Stress Test

The Seahorse XF96 Extracellular Flux Analyzer (Seahorse Bioscience, USA) was used to perform the glycolysis stress test per manufacture's protocol. For more detailed information please refer to Supporting Information.

### Glucose Consumption and Lactate Production

Glucose consumption and lactate production were measured by using the commercially available kits according to the manufacturer's instructions.

### Statistical Analyses

The independent samples *t*‐test was used for statistical analysis in triplicates of cell biology experiments. All data are presented as mean ± standard deviation. Statistic calculation was performed by SPSS 21.0 (IBM). A *p*‐value of <0.05 was considered statistically significant. Numbers of replicate experiments (*n*) are shown in figure legends. For all statistics, data from at least three independent samples or repeated experiments were used. All animals were allocated into experimental or control groups randomly.

## Conflict of Interest

The authors declare no conflict of interest.

## Author Contributions

X.Z., X.Z., Z.Z., J.D., G.F., and Y.H. performed the experiments and X.Z., X.Z., Z.Z., and X.C. analyzed the data. P.Z. designed the project. P.Z. wrote the manuscript. P.Z. and H.W. revised the manuscript.

## Supporting information

Supporting Information

## Data Availability

The data that support the findings of this study are available from the corresponding author upon reasonable request.
