# Supplementary material for: Small Molecule‐Induced Differentiation As a Potential Therapy for Liver Cancer
Source: Adv Sci (Weinh). 2022 Mar 27;9(15):2103619. doi: 10.1002/advs.202103619 (PMC9131429; doi:10.1002/advs.202103619)
Supplement: Supplementary file 1 — Supporting Information [file ADVS-9-2103619-s001.pdf]

ADVANCED SCIENCE
Open Access

Supporting Information

for *Adv. Sci.*, DOI 10.1002/advs.202103619

Small Molecule-Induced Differentiation As a Potential Therapy for Liver Cancer

*Xu Zhang, Xiang-Jie Zhu, Zhi Zhong, Jiang-Chuan Du, Guo-Xu Fang, Xiu-liang Cui, Ling-Ting Guan, Yan-Yu Hu, Hong-Yang Wang and Pei-Lin Zhang**

Small molecule-induced Differentiation as a Potential Therapy for Liver Cancer

Xu Zhang[1#], Xiang-Jie Zhu[1,3#], Zhi Zhong[1,3#], Jiang-Chuan Du[1], Guo-Xu Fang[1,4], Xiu-liang Cui[1,2], Ling-Ting Guan[1],Yan-Yu Hu[1], Hong-Yang Wang[1,2], Pei-Lin Zhang[1,2]**

# These authors contributed equally to this work.

** These authors jointly supervised this work.*

X.Zhang, X.Zhu, Z.Zhong, J.Du, X.Cui, L.Guan, Y.Hu, H.Wang, P.Zhang
National Center for Liver Cancer
Shanghai 201805, China
X.Cui, H.Wang, P.Zhang
Eastern Hepatobiliary Surgery Hospital, Shanghai 200438, China.
X.Zhu, Z.Zhong
Fudan University
Shanghai 200433, China.
G.Fang
Graduate School of Fujian Medical University,
Fuzhou, Fujian Province 350108, China

SUPPLEMENTARY MATERIALS

Table of Contents

Supplementary Materials and Methods……... 3

Fig. S1……………………………………………... 10

Fig. S2……………………………………………... 11

Fig. S3……………………………………………... 12

Fig. S4……………………………………………... 13

Fig.S5…………………………………………….. 14

Fig. S6……………………………………………... 15

Fig. S7……………………………………………... 16

Fig. S8……………………………………………... 16

Fig. S9……………………………………………... 17

Fig. S10……………………………………………... 18

Fig. S11……………………………………………... 19

Fig. S12……………………………………………... 19

Table 1……………………………………………... 20

Table 2……………………………………………... 22

Table 3……………………………………………... 23

Supplementary Material and Methods

Small molecules. Small molecules (SB431542, RepSox, CHIR99021, BIO, LY2090314, LDN193189, RG108, QNZ, Bix01294, VPA, Decitabine, TTNPB and Brdu) were purchased from Selleck biotechnology. ATRA and VitC were purchased from Sigma. AM580 was purchased from MedChemExpress (MCE). Please refer to Table1 for details.

Cell culture. HCC cell lines were purchased from ATCC and Cell Bank of Chinese Academy of Sciences (Shanghai, China), and cultured in Dulbecco's modified Eagle's medium (DMEM, High Glucose; Hyclone) supplemented with 10% fetal bovine serum (FBS; Biological Industries) and penicillin/streptomycin (100 units/ml and 100 μg/ml, respectively; Gibco) and maintained at 37°C in an atmosphere of humidified air containing 5% CO2. Medium was changed every two days and when cells growth reached about 80% confluent, cells were passage culture in a ratio 1:3-1:5 depending on the cell lineage.

Sorafenib resistant cell line establishment. Sorafenib resistant cell lines (Hep3B/Sora and HepG2/Sora) were established through stepwise selection of surviving Hep3B and HepG2 cells that are exposed to increasing doses of sorafenib (Sora) from 1 μM till final dosage of 15 μM.

Cell apoptosis detection. Cell apoptosis detection was performed according to the manufacturer's instructions (Dead Cell Apoptosis Kit, Invitrogen, Catalog No: V13245). Briefly, cells in each group were harvested using trypsin-EDTA solution and then washed with cold phosphate buffered saline (PBS). Approximately $1x10^5$ cells were collected in the single-cell solution. Binding buffer was added to suspend cells, which were stained with annexin V-FITC or propidium iodide (PI) at room temperature for 15 minutes in dark. Fluorescence intensities of the stained cells were measured by a Beckman FACS machine (Beckman, CY-355-100).

Cell growth ability evaluation. Cell proliferation assay was carried out using Cell Counting Kit-8 (CCK-8; Dojindo Laboratories). Cells of each group were seeded in 96-

well plates (1000~2000 cells/well) in triplicates. Cells were allowed to attach for 5~7 hours, and then medium were replaced with treatment addition. According to the manufacturer's instructions, CCK-8 reagent (1:100) was added into the test wells at the indicated time points (Day0, 1, 3, 5, 7). After an incubation of 30 minutes at 37°C, absorbance was measured at 450 nm using a microplate reader (BioTek Instruments).

Cell cycle detection. Cell cycle histograms were determined using PI staining by flow cytometry. Cells were plated in 6-well plate or 60 mm petri dish (20%~30% confluence) with triplicate, and then fresh medium were replaced containing DMSO or small-molecule compounds (SMC) after cells attached for 6~7 hours. Before reaching 80% confluence in 48~72 hours, cells were harvested by trypsinization, centrifuged and resuspended in 0.5 mL PBS. Following this, cells were then fixed within 5 mL ice-cold 70% ethanol overnight at 4°C. The pellets were collected by centrifugation and washed in PBS solution once. Finally, resuspended cells in PI staining solution (PI: 30 μg/mL, Beyotime; RNaseA: 100 μg/mL; 100%Triton X-100: 1 μL/mL, dissolved in PBS). After incubation for 30 minutes in the dark at room temperature, cells were analyzed for DNA content using a FACS machine (Beckman, CY-355-100). Cell distribution among cell cycle phases were evaluated using Summit software (Beckman).

Edu incorporation assay. According to the manufacturer's instructions, multiple procedures were carried out step by step. First of all, 10μM EdU (RiboBio, Guangzhou, China) was added to the culture media and maintained for 2-hour incubation for DNA labeling of cultured cells. To remove redundant EdU, cells were washed with PBS for 2~3 times carefully. And then, cells were harvested from each group into eppendorf tubes. After fixation with 4% paraformaldehyde for 10 min, cells were washed with 2 mg/mL glycine solution turning upside down the tubes for 5 min and rinsed once with PBS. Afterwards, cells were resuspended in 0.2% Triton X-100 (dissolved in PBS) for permeabilization, 10 minutes later, and cells were rinsed again with PBS. Next, freshly prepared 1× Apollo staining solution were added into the tubes and incubated for 30 min in the dark at room temperature. After staining, the cells were washed 2~3 times with

0.2% Triton X-100 supernatant was discarded carefully, methanol was used to wash cells for 5 min. Finally, cells were analyzed after PBS wash by FACS.

PAS staining, oil red o staining. The PAS and Oil Red O staining kits were purchased from Njjcbio. Cells were fixed with 4% paraformaldehyde in PBS and stained according to the manufacturer's instructions.

Immunofluorescent staining. Cells were blocked with PBS containing 0.2% Triton X-100 and 5% normal goat serum (NGS) at room temperature for 30 minutes and then incubated with anti-ALB, AAT, HNF4α and CYP3A primary antibodies for 1 hour at room temperature or at 4°C overnight. For the cell surface antigens ASGPR1 detection, only 5% NGS was used for blocking. After washed with PBS for three times, incubated with appropriate secondary antibodies for 30 minutes at room temperature in the dark. After incubation, cells were washed again and analyzed by FACS. Experiments were repeated for three times and typical results were shown. The primary antibodies are as follows: mouse anti CYP3A (sc-365415, Santa cruz biotechnology); mouse anti HNF-4α (sc-365415, Santa cruz biotechnology); mouse anti ASGPR1 (sc-52623, Santa cruz biotechnology); FITC-Conjugated polyclonal rabbit anti-human albumin (Nr. F 0117, Dako); mouse anti purified human alpha-1-antitrypsin (MA1-90438, Thermo Fisher). The secondary antibodies are as follows: goat anti-mouse, Alexa Fluor® 594 conjugate (R37121, Thermo Fisher); goat anti-rabbit, Alexa Fluor® 594 conjugate (R37117, Thermo Fisher).

The quantitative of albumin and urea production. Human albumin and urea assay kits were purchased from BioAssay Systems. Cells were seeded on 24-well plates for 48 hours and then medium were replace using phenol-free medium for additional 24 hours. Supernatant was collection for the albumin and urea detection. Due to the low concentration of ALB and urea in the samples, standards were diluted 10 fold to make sure the obtained values come within the linear range of standard curve. After the mixture of working reagent and samples in duplicate, incubated 5 min at room temperature and read optical density (OD) at 620nm for ALB assay, whereas 50-min incubation and

430nm OD value were suitable for urea assay. An extra well of total cell DNA content was used to normalize the data.

The induction of CYP metabolism activity. To induce CYP metabolism activities, cells were cultured in 12-well plate for 24 hours and then replaced medium supplemented with chemical inducers (omeprazole for CYP1A2, rifampicin for CYP3A4) daily for 48 hours, then total RNA was isolated and CYP gene expression level was quantitative using real-time RT-PCR assay.

Plate colony formation assays. For plate colony formation assay, 1,000 cells were counted and seeded in 6-well plates in triplicate, and cultured in 2~2.5 mL medium for 10~15 days. Cells were fixed and crystal violet solution was added for staining for 10 minutes, then the staining was rinsed with ddH2O. Images were taken using regular camera.

Migration and invasion assay. Cells were counted to reach almost 100% confluent and seeded on polycarbonate transwell membrane (Greiner Bio-One) which match 24-well plate. After the cells attached to the membrane firmly (6~8 h), replaced fresh DMEM medium with 1% FBS inner and 20% FBS outside of insert. Cells were fix and stained after a certain period according to the variations of different cells' migration ability. The distinction between the two assays are the transwell membrane in invasion assay was previously coated with 20% Matrigel (356231, Corning) and the waiting time was prolonged either.

Primary HCC patient derived cancer cells isolation and culture. Tumor tissue that derived from HCC patients were carefully dissected to remove necrotic tissue, connective tissue and blood vessels. After tumor tissue was cut into small pieces, about 10 ml collagenase type IV solution was directly added to the tumor for the digestion in 37°C for 20~30 minutes. After blowing up-down for several times using pipette, the collagenase solution containing tumor cells was passed the sterile 70μm nylon filters to collect single-cell solution. Cells were centrifuged and washed using culture medium for 3 times, cell

yield and viability were assessed through trypan blue exclusion. Fresh isolated tumor cells were cultured in DMEM with 10% FBS, 1% ITS supplement, 20ng/ml EGF addition. Medium were changed every two days.

Xenograft Studies: Mice were manipulated and housed according to the criteria outlined in the "Guide for the Care and Use of Laboratory Animals" prepared by the Eastern Hepatobiliary Surgery Institute. All xenograft experiments were approved by the Research Ethics Committee of Eastern Hepatobiliary Surgery Institute.

***In vivo* tumor formation experiment.** BALB/C male nude mice were purchased from Sino-British SIPPR/BK Lab Animal Ltd. Control and treated cells (1×10^6) were injected subcutaneously into the flanks of mice. Control cells were injected into left side while treated cell in right side (back view). When the tumor grew up to reach 1 cm3, mice were sacrificed and images were taken. The tumors were dissected for other experiment usage.

Cell-derived xenograft (CDX) mouse model generation and treatment. 6-8 weeks old BALB/C male nude mice were purchased from Sino-British SIPPR/BK Lab Animal Ltd. **Subcutaneous CDX model:** Approximately $1x10^6$ mCherry-Luciferase labeled Hep3B cells were suspended in 50μL of DMEM and Matrigel (BD Bio-sciences) (1:1) and injected subcutaneously into two legs of the nude mice. After tumor sizes reached 5-9×5-9mm, animals were randomly divided into two groups, and received control and SMC treatment through intra-tumor injections, respectively. **Orthotopic CDX model:** approximately $1x10^6$ Hep3B or Hep3B/Sora cells labeled with mCherry-Luciferase were suspended in 20μL Matrigel and injected into mice livers. 20 days after HCC cell injection, the viability of cells was monitored based on luciferase activity through *in vivo* imaging. Once tumor formation is confirmed, animals were randomly divided into two groups and treated with SMC and control condition by oral administration daily.

Liver tumor patient-derived xenograft (PDX) mouse model generation and treatment. All HCC patients and their relatives provided informed consents, and the study was approved by the ethics committee of Eastern Hepatobiliary Surgery Institute.

Tumor samples were maintained in Dulbecco's modified Eagle's medium containing 10% fetal bovine serum, penicillin, streptomycin for less than 2 hours at room temperature before implantation. **Subcutaneous PDX model:** The tumor samples were dissected into fragments ranged between 2~3 mm, and tissue in the size of 3×3 mm was injected subcutaneously into the flanks of 5-week-old BALB/C male nude mice (obtained from Sino-British SIPPR/BK Lab. Animal Ltd, Shanghai, China). After tumor growth reached 5-9×5-9mm, the tumors were isolated and the same preparation process as above was repeated, then tumor tissue was transplanted into the flanks of 5-week-old BALB/C male nude mice as P1 PDX tumors. After transplantation of P3 PDX tumor tissues, when tumor growth reached to 5x5mm, the animals were randomly allotted to two groups, and received SMC or control treatment, respectively. Tumor size was monitored every week. SMC and control solvent were freshly prepared and injected into tumor mass over a period of 4 weeks (3 times a week). **Orthotopic PDX model:** P3 PDX tumors were dissected into fragments ranged from 1~2 mm. Approximately 2-4 tumor fragments (generated from the same P1 PDX tumor) were implanted under hepatic subcapsular regions of NOD/SCID mice (purchased from Sino-British SIPPR/BK Lab Animal Ltd). 4 weeks after tumor tissue implantation, animals were randomly divided into two groups and treated with SMC and control condition by oral administration daily for 4 weeks.

Liver tumor metastatic mouse model generation and treatments. 6-8 weeks old male nude mice were purchased from Chinese Science Academy (Shanghai, China). HCC cell LM3 labeled with mCherry-Luciferase were suspended in 100μL NaCl and injected into 6-8 weeks old male nude mice's spleen. 20 days after HCC cell injection, the viability of cells was monitored based on luciferase activity through *in vivo* imaging. Once tumor formation is confirmed, animals were randomly divided into two groups and treated with SMC and control condition by oral administration daily for 4 weeks.

Quantitative RT–PCR. Total RNA was isolated from cells by TRIzol (Invitrogen) in the standard protocol, and reverse transcription was performed using M-MLV Reverse Transcriptase (Invitrogen) by using 2μg RNA. Quantitative PCR was performed using a Roche Light Cycler 96 System (Roche, USA) and Sybr Green Supermix (Bio-Rad

Laboratories). The following conditions were used for qRT-PCR: 5 min at 95°C, and 40 cycles of 15 sec at 95°C and 30 sec at 55°C. Reactions were carried out in triplicate with the delta Ct method. Please refer to Table 2 for details on qRT-PCR primers sequences.

SDS–PAGE and western blot analysis. Whole-cell lysates were run on 10% SDS-polyacrylamide gels and transferred to nitrocellulose membrane (GE) by standard methods. Membranes were blocked for 1~2 hours in 5% BSA in 1×TBS with 0.1% Tween-20 (TBST) and incubated with primary antibody diluted in 5% BSA in TBST overnight at 4°C. The information of primary antibodies, please refer to Table 3 for details. After washing blots in TBST for 3 times, IRDye 800CW Secondary Antibodies (LI-COR) were incubated in 5% BSA in TBST for 1 hour at room temperature. Fluorescent infrared signal was detected using LI-COR Odyssey imaging system.

Mitochondrial stress test and Glycolysis stress test: The Seahorse XF96 Extracellular Flux Analyzer (Seahorse Bioscience, USA) was used to perform the glycolysis stress test per manufacture's protocol. Cells (1 × 104 per well) were maintained for 24 h at 37°C in a 5% CO2 incubator. Extracellular acidification rate (ECAR) was measured prior to and after injection of 10 mM glucose, 2 mM oligomycin and 50 mM 2-DG. Data were analyzed using Wave 2.1 and normalized to protein concentration.

Glucose consumption and lactate production. Glucose consumption and lactate production, were measured by using the commercially available kits according to the manufacturer's instructions.

Statistical analyses. The independent samples t-test was used for statistical analysis in triplicates of cell biology experiments. All data are presented as mean ± standard deviation. Statistic calculation was performed by SPSS 21.0 (IBM). A P value of <0.05 was considered statistically significant. Numbers of replicate experiments (n) are shown in figure legends. For all statistics, data from at least 3 independent samples or repeated experiments were used. All animals were recruited into experimental or control groups randomly.

Supplementary Figure Captions

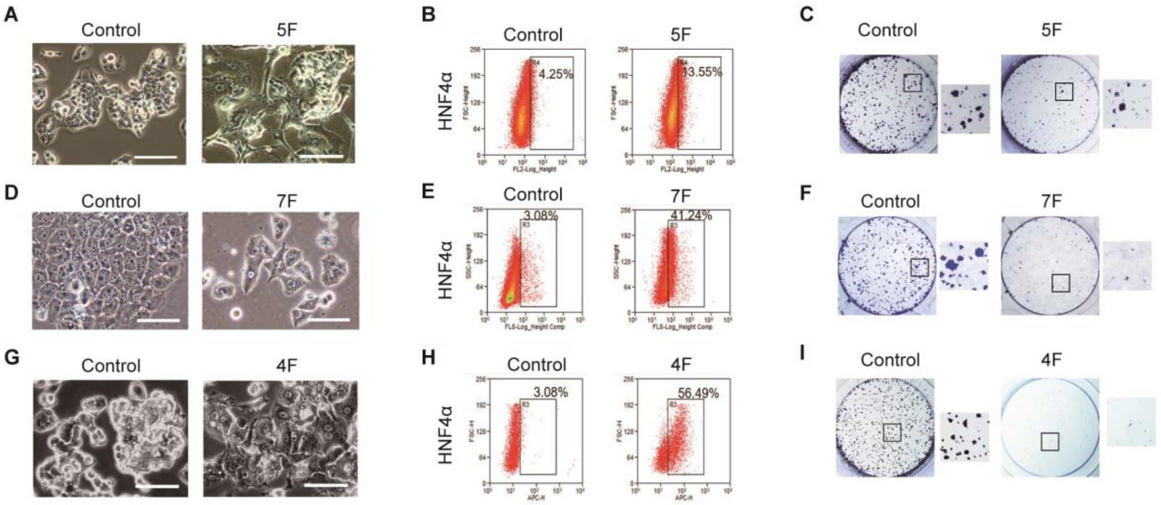

Figure S1. Ability of different combination of chemicals to induce hepatic conversion of HepG2 cells.

(A) Representative morphologies of HepG2 cells treated with small molecules combination SCVZA (5F) for 14 days. Most cells' morphology reverted back to normal, but some still retained tumor cell morphology.

(B) FACS analysis of hepatocyte-specific marker HNF4α expression in HepG2 cells treated with small molecules combination SCVZA (5F) for 14 days.

(C) Colony formation ability of HepG2 cells treated with 5F for 14 days. Few colonies emerged.

(D) Representative morphologies of HepG2 cells treated with 7F (SCVZABBr) for 14 days. Almost all cells gained hepatocyte's morphology.

(E) FACS analysis of HNF4a expression in HepG2 cells treated with 7F for 14 days.

(F) Colony formation ability of HepG2 cells treated with 7F for 14 days. No colony was observed.

(G) Representative morphologies of HepG2 cells treated with SCBA (4F) for 14 days. Almost all cells gained hepatocyte's morphology.

(H) FACS analysis of HNF4a expression in HepG2 cells treated with 4F for 14 days.

(I) Colony formation ability of HepG2 cell treated with 4F for 14 days. No colony was observed.

5 factor cocktail (SCVZA): SB431542 (S), CHIR-99021(C), VPA (V), 5-AZA (Z) and ATRA (A).

7 factor cocktail (SCVZABBr): SB431542 (S), CHIR-99021(C), VPA (V), 5-AZA (Z), ATRA (A), BIX01294(B), Brdu(Br).

SMC - 4 factor cocktail (SCBA): SB431542 (S), CHIR-99021(C), BIX01294 (B) and ATRA (A).

Scale bar: 100μm.

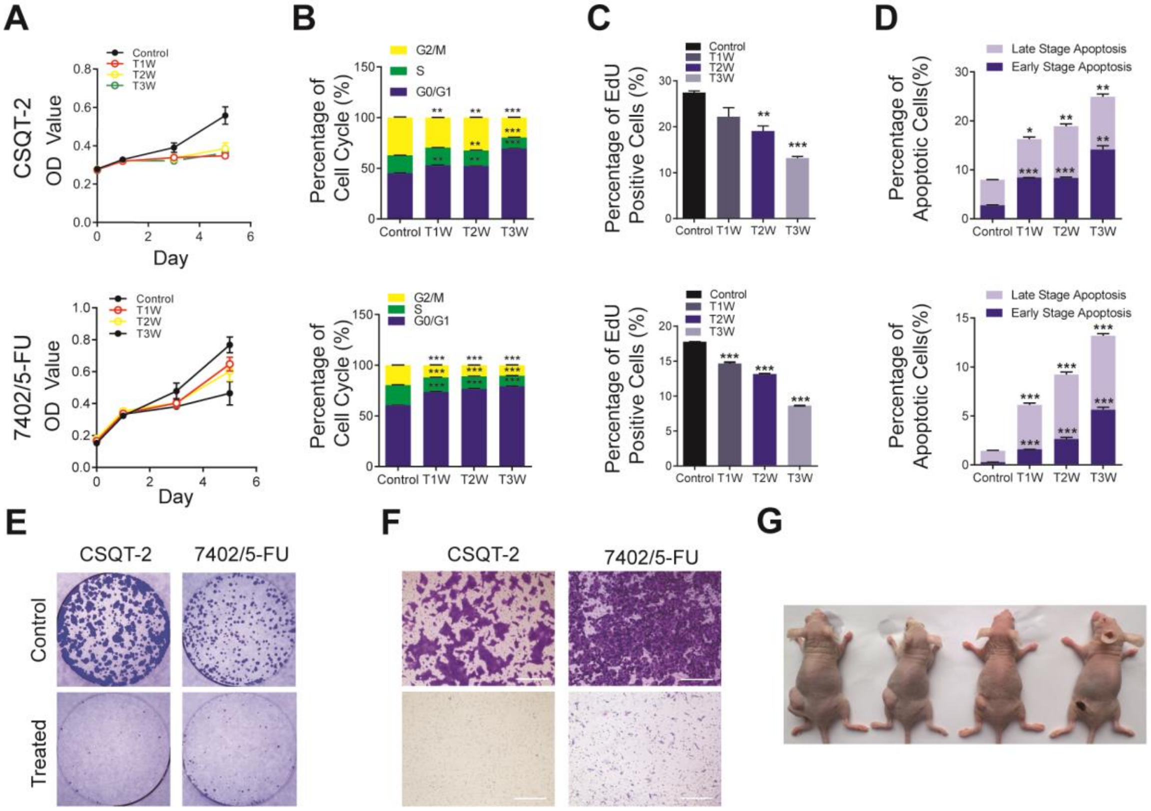

Figure S2. SMC-treated HCC cells lose malignant features and tumorigenicity *in vitro* and *in vivo*

(A) Relative cell viability of SMC-treated CSQT-2 and BEL-7402/5-FU (HCC) cells measured by CCK-8 assay at indicated time points.

(B) Cell cycle analysis of SMC-treated HCC cells using PI staining and flow cytometry.

(C) FACS analysis of EdU incorporation rate of SMC-treated HCC cells.

(D) FACS analysis of apoptotic HCC cell population after SMC treatment measured with annexin V staining.

(E) Plate colony formation assays of SMC-treated HCC cells.

(F) SMC treatment led to reduced cell migration ability in HCC cells.

(G) In vivo tumorigenicity test: tumor formation in nude mice engrafted with SMC-treated and control BEL-7402/5-FU cells. Control cells were injected into the left dorsal flanks of mice, and SMC-treated cells were injected into the right dorsal flanks.

HCC Cells: CSQT-2 and BEL-7402/5-FU cell lines. T1W, T2W and T3 W represent SMC treatment for 1, 2 or 3 weeks.

Data represent the means ± SD. Asterisks indicate significance compared to control untreated cells as assessed by t-test: *$p < 0.05$; **$p < 0.01$; ***$p < 0.001$.

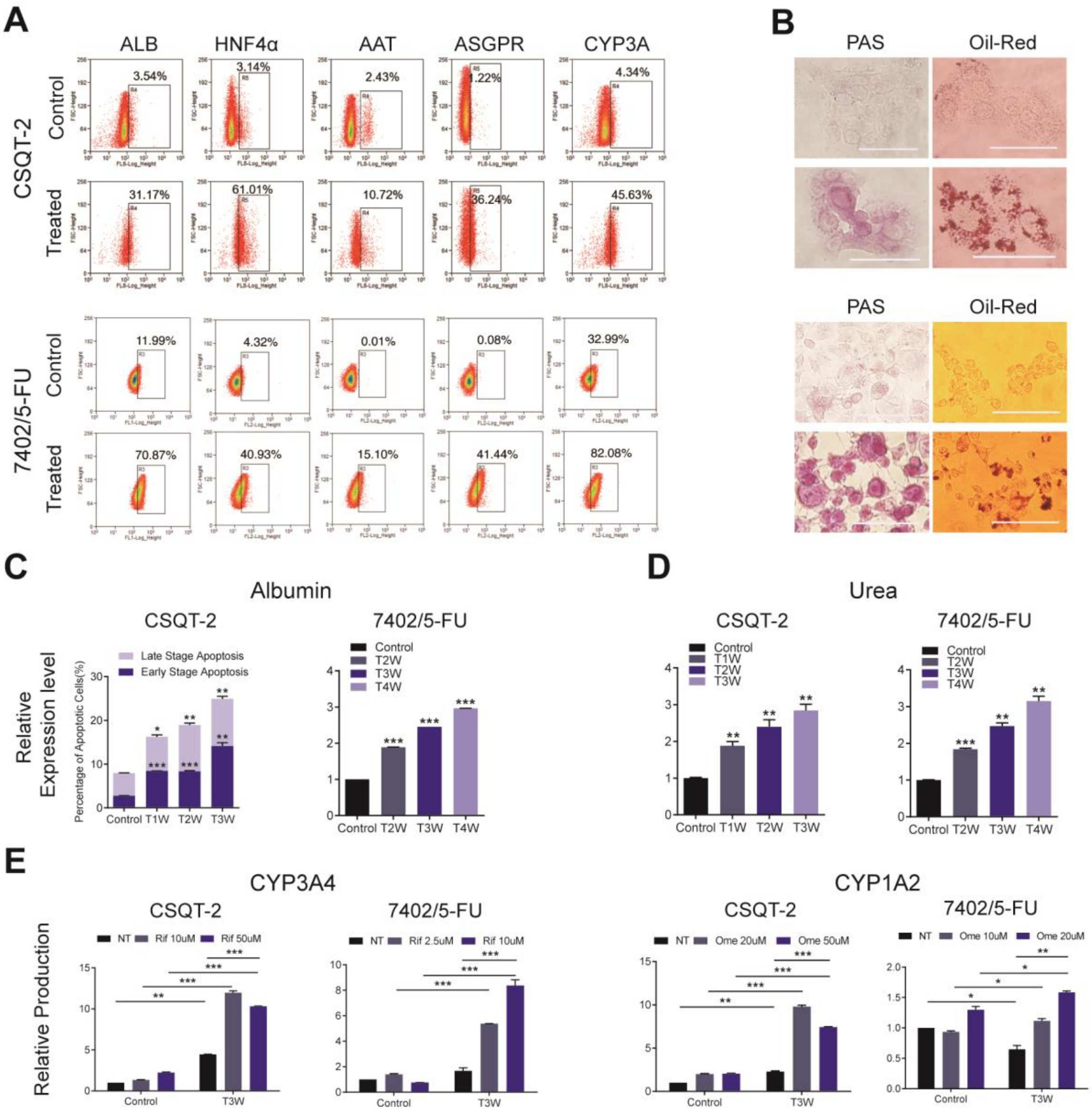

Figure S3. SMC-treated HCC cells gain hepatic cell features and functions.

(A) Expression of hepatocyte-specific markers in CSQT-2 and BEL-7402/5-FU (HCC) cells treated with SMC for 21 days (3 weeks) measured by FACS analysis. Primary human hepatocytes (PHH) were used as positive control.

(B) Cytoplasmic accumulations of glycogen and lipid by HCC cells treated with SMC for 21 days (3 weeks) were determined by PAS and oil-red O staining.

(C and D) Relative ALB and urea production of HCC cells treated with SMC for 1, 2 and 3 weeks. ALB and urea production of PHH sample were positive control.

(E) PAS and oil-red O staining of PHHs as positive controls.

(F and G) qPCR measured mRNA levels of CYP genes before and after drug induction in HCC cells

treated with SMC for 21 days. CYP3A4 and CYP1A2 were induced by the indicated concentrations of rifampicin and omeprazole, respectively, for 48 hours. Untreated HCC cells were used as controls. Rif, rifampicin; Ome, omeprazole.

HCC Cells: CSQT-2 and BEL-7402/5-FU cell lines. T1W, T2W and T3W represent SMC treatment for 1, 2 or 3 weeks.

Data represent the means ± SD. Asterisks indicate significance as assessed by t-test: *$p < 0.05$; **$p < 0.01$; ***$p < 0.001$.

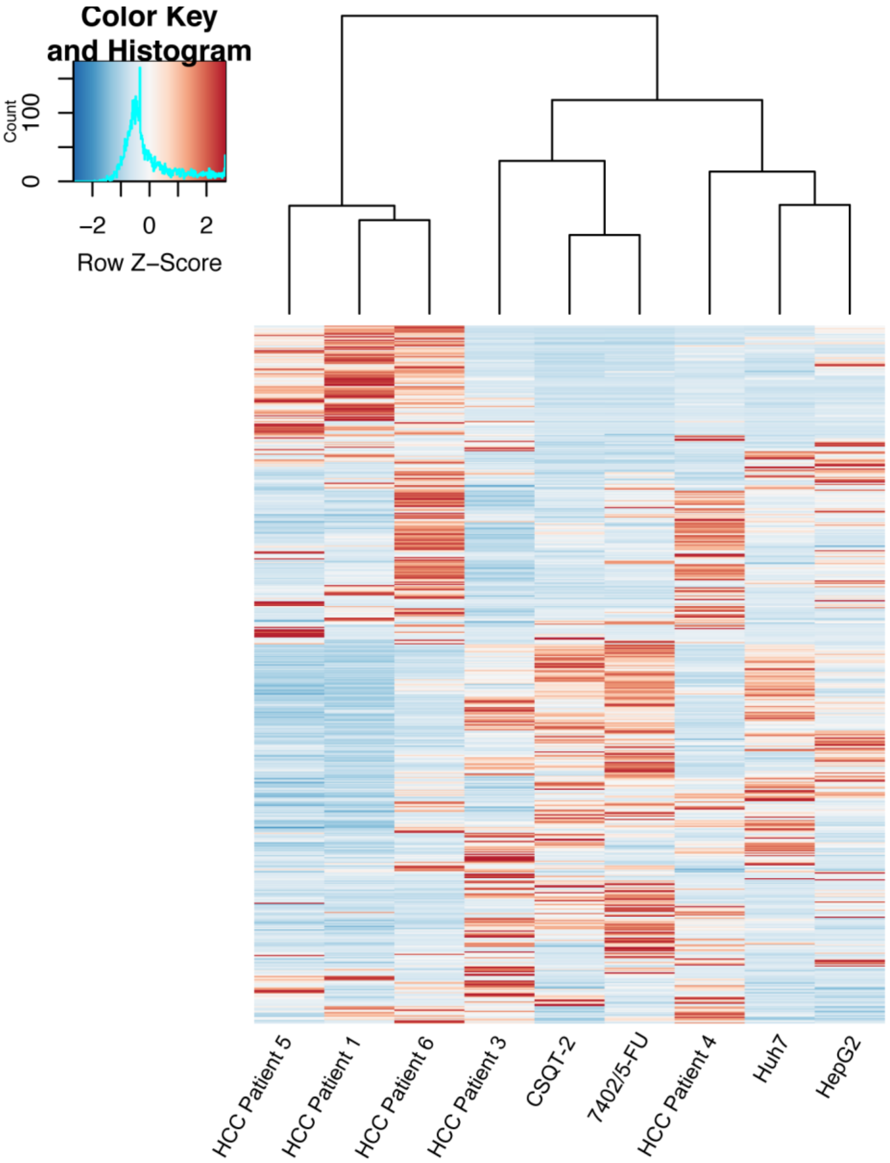

Figure S4. The RNA-seq results of different liver cancer cells to demonstrate the heterogeneity.

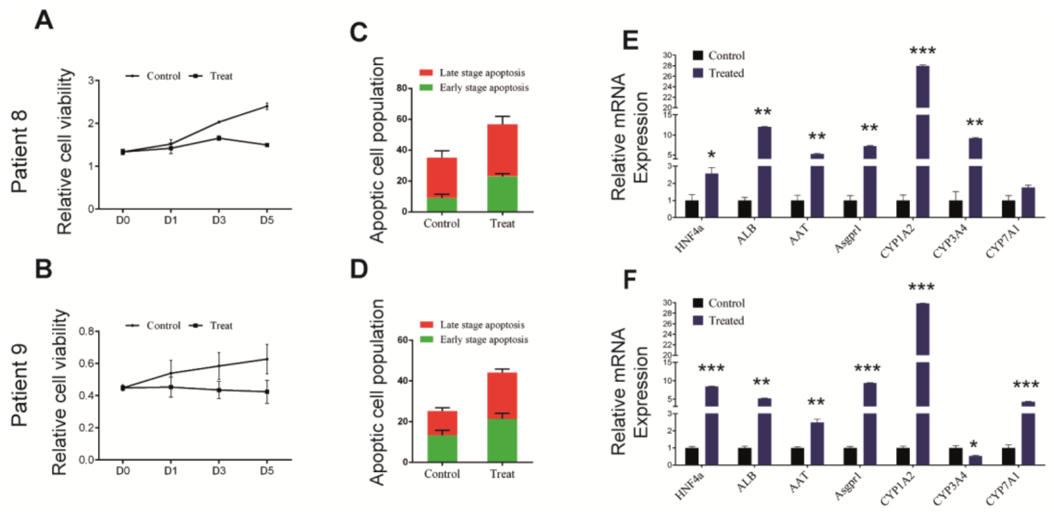

Figure S5. SMC induce patient-derived liver tumor cell differentiation.

(A-B) Relative cell viability of control and SMC-treated human primary liver tumor cells measured by CCK-8 assay (n=3).

(C-D) FACS analysis of apoptotic liver tumor cell population measured by annexin V staining (n=3).

(E-F) Relative mRNA expression of hepatocyte-specific genes in SMC treated or control cells (n=3).

A, C and E results derived from patient 8, B, D and F results derived from patient 9.

Data represent the means ± SD. Asterisks indicate significance compared to control untreated cells as assessed by t-test: *$p < 0.05$; **$p < 0.01$; ***$p < 0.001$.

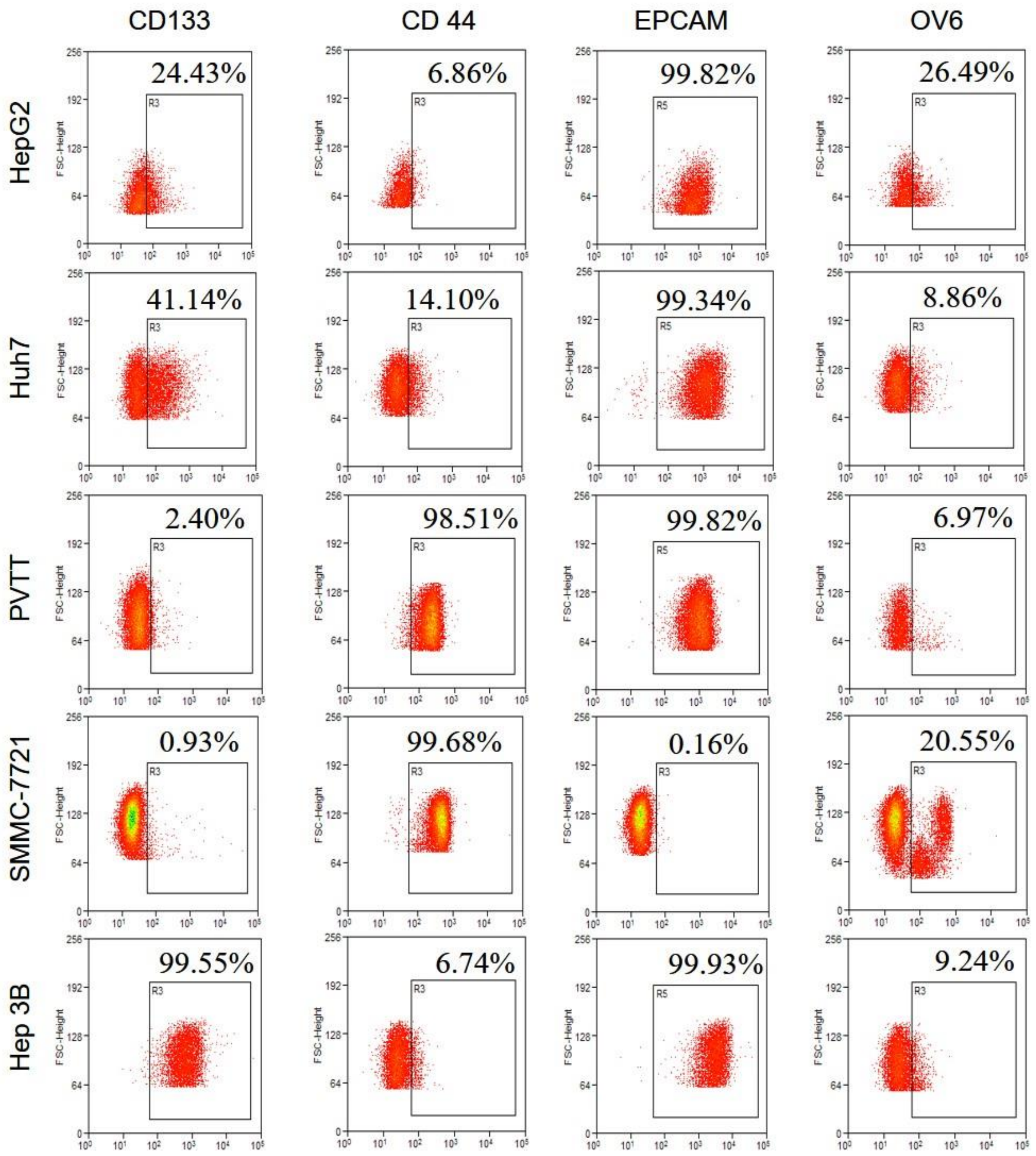

Figure S6. Expression of signature CSC markers in different HCC cell lines by FACS analysis. CD133, CD44, EPCAM and OV6: CSC markers; HepG2, Huh7, CSQT-2, SMMC-7721 and Hep3B: HCC cell lines.

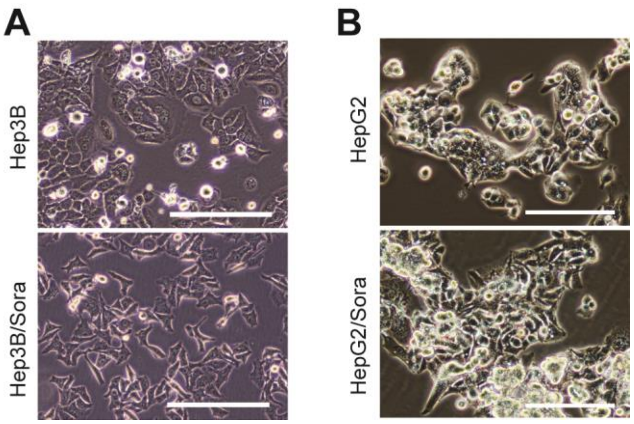

Figure S7. Phenotype of Sorafenib-resistant HCC cell lines Hep3B/sora and HepG2/sora in comparison with their parental cells Hep3B and HepG2.

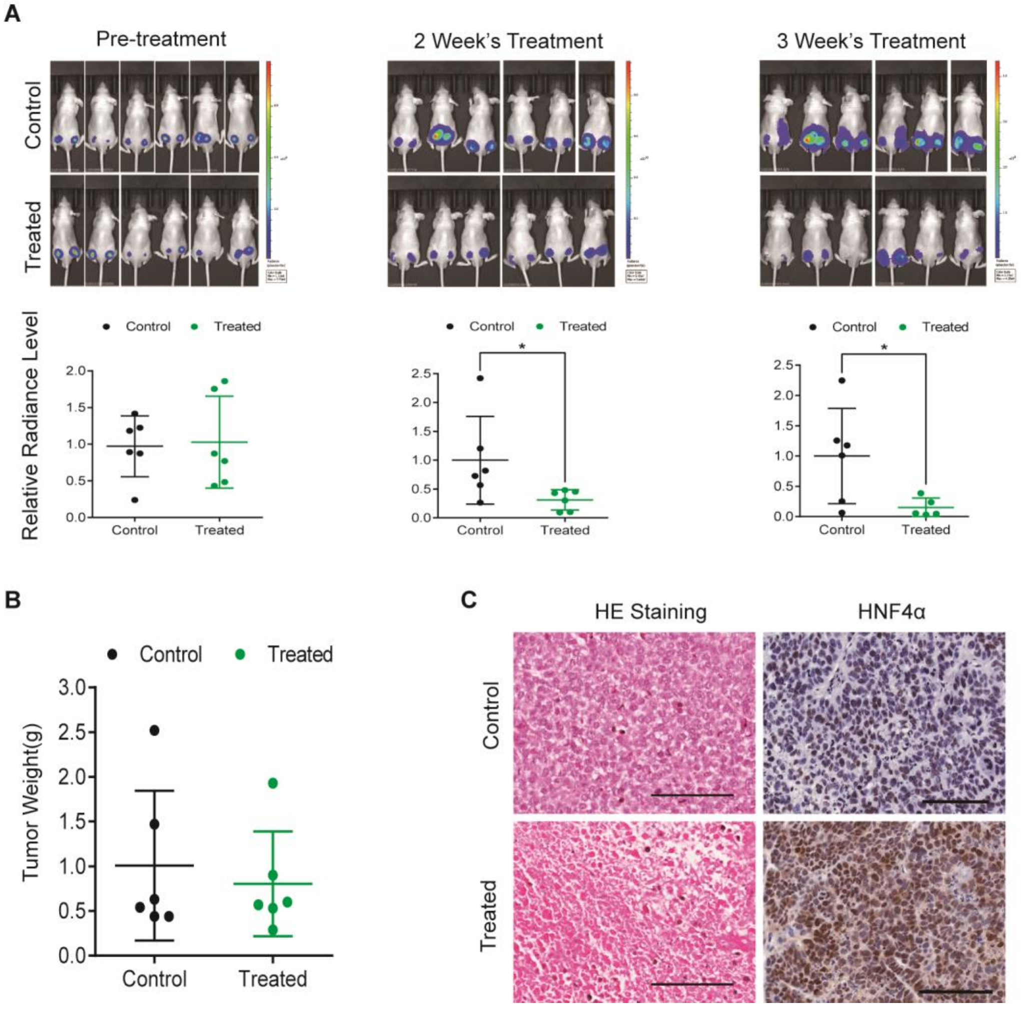

Figure S8. Anti-tumor effect of SMC on HCC CDX Model.

(A-B) Radiance intensity of SMMC-7721 cells on CDX mice model treated with SMC through intra-tumor injection measured by in vivo imaging. Upper lane: IVIS image, lower lane: Statistic analysis (n=6).

(C) Dissected tumors from mice after 4 weeks of treatment with control and SMC, respectively.

(D) Representative photomicrographs of HE staining showing tissue necrosis in tumors dissected from SMMC-7721 CDX models treated with SMC for 4 weeks. The remaining tumor tissue had positive HNF4α staining (n=6).

Scale bar, 100 μm. Data represent the means ± SD. Asterisks indicate significance as assessed by t-test: *p < 0.05, **p < 0.01, ***P < 0.001

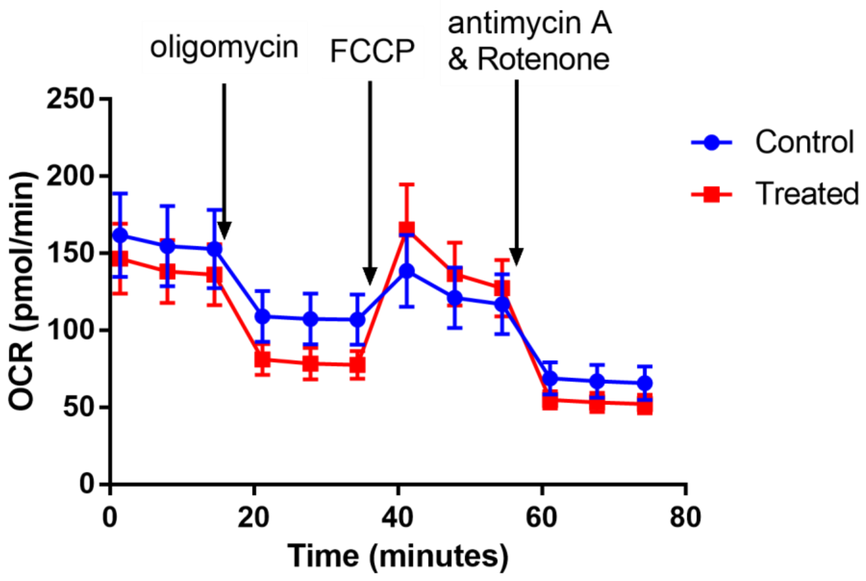

Figure S9. Oxygen Consumption Rate (OCR) in control and SMC-treated HepG2 cells on day 10 of culturing measured by XF96 Extracellular Flux Analyzer.

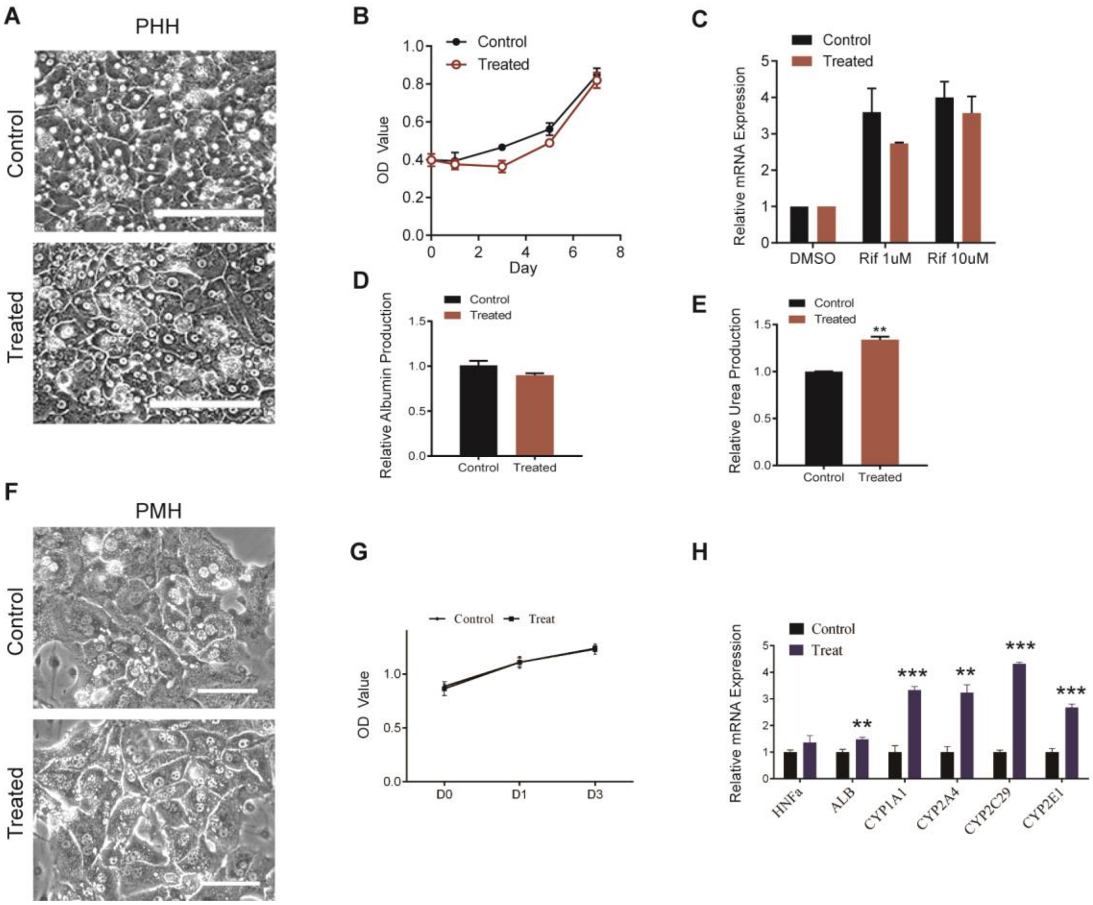

Figure S10. The effect of SMC on normal primary hepatocytes.

(A) Cell morphology comparison of control and SMC-treated primary human hepatocytes (PHHs).

(B) Relative cell viability of control and SMC-treated PHHs measured by CCK-8 assay (n=3).

(C) qPCR measured mRNA expression of CYP3A4 genes in control and SMC-treated PHHs before and after rifampicin induction for 48 hours. Rif, rifampicin.

(D-E) Relative albumin production (D) and urea synthesis (E) by control and SMC-treated PHHs.

(F) Cell morphology of control and SMC-treated primary mouse hepatocytes (PMHs).

(G) Relative cell viability of control and SMC-treated PMHs measured by CCK-8 assay (n=3).

(H) Relative mRNA expression of hepatocyte-specific genes in control and SMC-treated or control PMHs (n=3).

Scale bar, 50 μm: Data represent the means ± SD. Asterisks indicate significance compared to control untreated cells as assessed by t-test: *$p < 0.05$; **$p < 0.01$; ***$p < 0.001$.

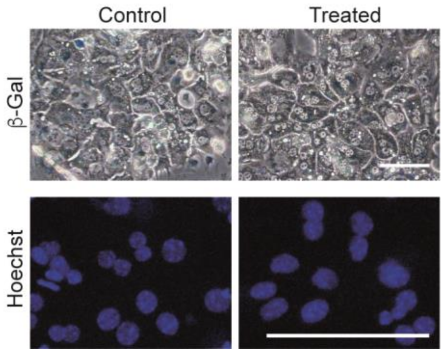

Figure S11. The effect of SMC on apoptosis and senescence of normal primary hepatocytes Representative images of β-Gal (beta-galactosidase) (for cell senescence) and Hoechst (for cell apoptosis) staining in control and SMC-treated primary mouse hepatocytes (PMHs).

Scale bar, 50 µm.

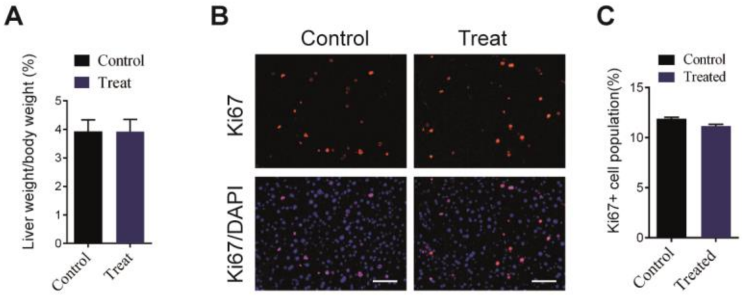

Figure S12. The effect of SMC on liver regeneration in 70% hepatectomy model

(A) The ratio of liver weight to body of mouse that suffered from 70% hepatectomy and treated with or without SMC for 7 days (n=6).

(B-C) Immunofluorescence staining of Ki67 in 70% hepatectomy liver tissue slices (B) and the quantification (C).

Scale bar, 100 µm.

Supplementary Table 1: Small molecules information

Full Name	Source	Molecular weight	Structure	Dosage/Concentration Used
SB431542	Selleck, Cat. No. S1067	384.39	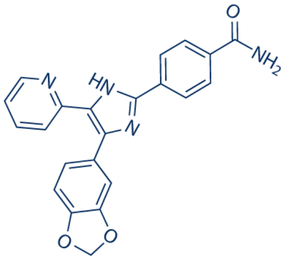	2μM(in vitro)<br>2mg/kg(in vivo)
RepSox	Selleck, Cat. No. S7223	287.32	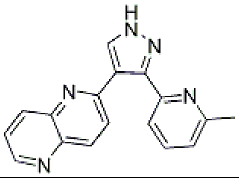	2-10μM
CHIR-99021	Selleck, Cat. No. S2924	501.8	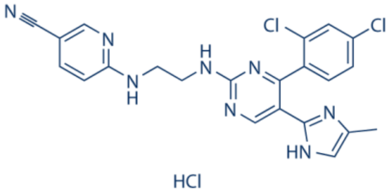	2-5μM(in vitro)<br>1mg/kg (in vivo)
BIO	Selleck, Cat. No. S7189	356.17	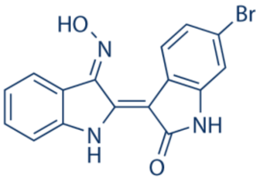	1-5μM
LY2090314	Selleck, Cat. No. S7063	512.53	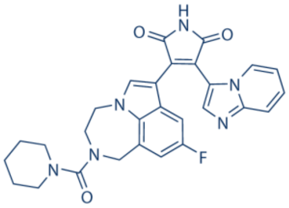	1-5μM
LDN193189	Selleck, Cat. No. S2618	406.48	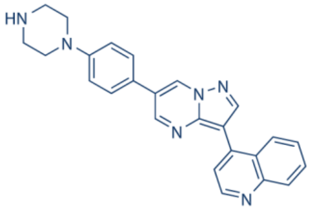	0.1-0.2μM
RG108	Selleck, Cat. No. S2821	334.33	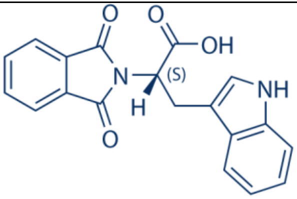	1μM

QNZ (EVP4593)	Selleck, Cat. No. S4902	356.42	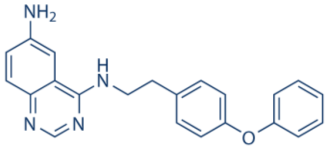	0.1-0.5μM
Bix01294	Selleck, Cat. No. S8006	600.02	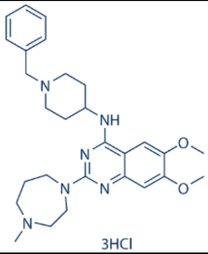	2.5μM(in vitro)<br>1mg/kg (in vivo)
VPA (Valproic acid)	Selleck, Cat. No. S3944	144.21	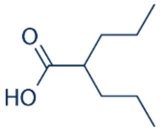	10μM
Decitabine (5-aza-CdR)	Selleck, Cat. No. S1200	228.21	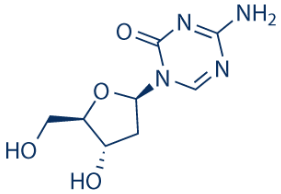	0.1-0.2μM
TTNPB	Selleck, Cat. No. S4627	348.48	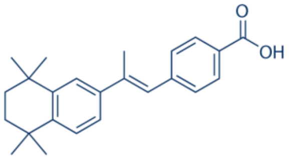	1-5μM
AM580	MCE, Cat. No. HY10475	300.44	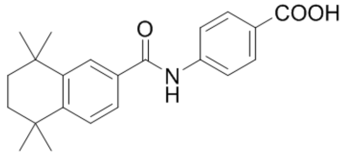	0.1-0.5μM
ATRA	Sigma, Cat. No. 302-79-4	334.33	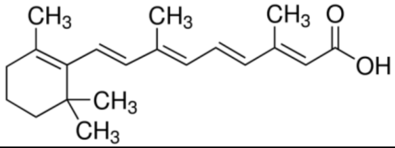	5μM(in vitro)<br>1mg/kg (in vivo)
Brdu	Selleck, Cat. No. S7918	307.1	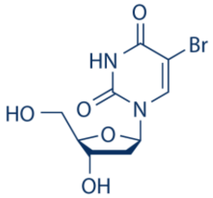	5-10μM
L-Ascorbic acid (VitC)	Sigma A7506	176.12	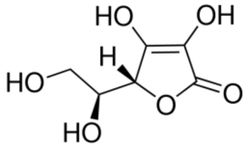	10-100μg/ml

Supplementary Table 2: Antibodies Information

**Antibody name**	**Company**	**Catalog Number**
**Antibodies for Western Blot**		
Rabbit Anti-gamma Tubulin antibody - Centrosome Marker (ab16504)	abcam	ab16504
HIF-1 alpha Antibody (H1alpha67)	NOVUSBIO	NB100-105
Phospho-4E-BP1 (Thr37/46) (236B4) Rabbit mAb #2855	CST	2855T
Phospho-mTOR (Ser2448) (D9C2) XP® Rabbit mAb #5536	CST	5536T
Phospho-p70 S6 Kinase (Thr389) (108D2) Rabbit mAb #9234	CST	9234T
c-Myc/N-Myc (D3N8F) Rabbit mAb #13987	CST	13987S
Phospho-Akt (Ser473) Antibody #9271	CST	9271S
LDHA Antibody #2012	CST	2012S
AFP (D12C1) Rabbit mAb #4448	CST	4448S
GLUT1 Antibody	Affinity Biosciences	AF0173
Glut 4 Antibody	Affinity Biosciences	AF5386
Hexokinase 2 Monoclonal Antibody	proteintech	66974-1-Ig
PFKFB3 (D7H4Q) Rabbit mAb #13123	CST	13123S
PKM2 (D78A4) XP® Rabbit mAb #4053	CST	4053T
Pyruvate Dehydrogenase (C54G1) Rabbit mAb #3205	CST	3205T
Snail (C15D3) Rabbit mAb #3879	CST	3879S
**Antibodies for Immunostaining – Primary Antibodies**		
HNF4a Monoclonal Antibody	R&D Systems	MAB4605
Polyclonal Rabbit Anti-Human Albumin	Dako	F0117
alpha-1 Antitrypsin Monoclonal Antibody	Thermo	MA5-14661
Asgpr1 Monoclonal Antibody	R&D Systems	MAB4394-100
CYP3A Monoclonal Antibody	Thermo	MA5-17064
**Antibodies for Immunostaining – Secondary Antibodies**		
Goat anti-Mouse IgG (H+L), Alexa Fluor Plus 488	invitrogen	A11029
Goat anti-Mouse IgG (H+L), Alexa Fluor Plus 594	invitrogen	A11005

Supplementary Table 3: Primers information

Primer name	Forward(5'-3')	Reverse(5'-3')
β-actin	GGCCAACCGCGAGAAGATGA	CCGGAGTCCATCACGATGCC
GAPDH	AAATCCCATCACCATCTTCC	ATGACCCTTTTGGCTCCC
ALB	CGCCTGAGCCAGAGATTTCC	CGCCCTGTCATCAGCACATT
AAT	GGAGATGCTGCCCAGAAGAC	GCATTGCAAAGGCTGTAGCG
Asgpr1	TGGCTCAGAAAGGACCTGCT	GCCCATCCAGGTGTTCACAG
HNF4α	CCCATCAGAAGGCACCAACC	TCTTTGTCCACCACGCACTG
CYP1A2	TGGGCACTTCGACCCTTACA	AGGCAGTCTCCACGAACTCA
CYP3A4	TTCAGCAAGAAGAACAAGGACAA	GGTTGAAGAAGTCCTCCTAAGC
CYP7A1	TGGGCACAGAAGCATTGACC	CAGGCAGCGGTCTTTGAGTT
AFP	TGTAGCGCTGCAAACGATGA	CTCCCAAAGCAGCACGAGTT
TTR	TAGATGCTGTCCGAGGCAGT	TCAGTTGTGAGCCCATGCAG
PTEN	GGCGGAACTTGCAATCCTCA	CTTGTCTTCCCGTCGTGTGG
TP53	AGTGTGGTGGTGCCCTATGA	CGCCCATGCAGGAACTGTTA
c-Myc	GTATGTGGAGCGGCTTCTCG	TGCAGGTACAAGCTGGAGGT
Oct4	GCAGATCAGCCACATCGCCC	GCCCAGAGTGGTGACGGAGA
Sox2	GTGAGCGCCCTGCAGTACAA	GCGAGTAGGACATGCTGTAGGTG
Nanog	CCTGTGATTTGTGGGCCTGA	CTCTGCAGAAGTGGGTTGTTTG
Glut1	GGCTTCTCGAAACTGGGCAA	ACATACATGGGCACGAAGCC
Glut4	ATGCCACCATAGGAGCTGGT	AGGAGCAGAGCCACAGTCAT
HIF1α	AGAAAGCAGTTCCGCAAGCC	TCAGTGGTGGCAGTGGTAGT
PKM2	TGGACCTCCGGGTGAACTTT	CGGTCAGCACAATGACCACA
LDH	ATTGGGCAAAGGCTTGGCAT	CTTGAGTCGCCATGCTCTCC
AMPK	TGTCACAGGCATATGGTGGTC	TGGGTGAGCCACAACTTGTTC
